# Artificial Intelligence in Cardiology Examination: A Comparative Study of Physicians, ChatGPT-4, and ChatGPT-5

**DOI:** 10.7759/cureus.106648

**Published:** 2026-04-08

**Authors:** Weronika Majchrowicz, Dawid Boczkowski, Aleksandra Wielochowska, Piotr Sawina, Anna Kowalczyk, Michalina Loson-Kawalec, Aleksander Tabor, Dawid Szymanski, Maciej Majchrzak, Mateusz Podkanowicz, Oliwia Mencel, Piotr Lukjanowicz, Michalina Wecka, Tomasz Dolata, Dawid Bartosik, Aleksander Polus

**Affiliations:** 1 Internal Medicine, Multispecialty Independent Public Health Care Institution Hospital in Nowa Sól, Nowa Sól, POL; 2 Pediatric Surgery, Central Teaching Hospital of Medical University of Łódź, Łódź, POL; 3 Psychiatry, Central Teaching Hospital of Medical University of Łódź, Łódź, POL; 4 Internal Medicine, Non-public Health Care Institution (NZOZ) Hospital in Dzierżoniów, Dzierżoniów, POL; 5 Faculty of Medicine, University of Opole, Opole, POL; 6 Faculty of Medicine, University of Zielona Góra, Zielona Góra, POL; 7 Internal Medicine, Central Teaching Hospital of Medical University of Łódź, Łódź, POL

**Keywords:** artificial intelligence, cardiology, clinical reasoning, education, gpt, language models, medical, specialty certification

## Abstract

Background

Medicine is increasingly applying artificial intelligence (AI), particularly large language models (LLMs). Previous studies have shown that models such as GPT-4 can achieve performance comparable to physicians on medical knowledge tests. However, direct comparisons between the newest GPT-5 model, its predecessor GPT-4, and physicians are lacking, especially with respect to theoretical versus clinical question types.

Methodology

This comparative study analyzed 120 multiple-choice questions from the spring 2025 Polish National Cardiology Specialization Examination administered by the Medical Examination Center (CEM) in Łódź. The dataset included 62 theoretical questions and 58 clinical scenario-based questions. Examination performance of physicians who sat for the exam (n = 153) was compared with responses generated by ChatGPT-4 and ChatGPT-5. Both models were evaluated independently using the original Polish-language questions under standardized, exam-like conditions, without access to external databases or tools. Model accuracy was calculated overall and by question type. Statistical comparisons were performed using one-sample proportion Z-tests and McNemar’s tests, with a Bonferroni correction applied for multiple comparisons (significance threshold set at p-values <0.0167).

Results

Physicians achieved a mean accuracy of 72.5% compared with 77.5% for ChatGPT-4 and 79.2% for ChatGPT-5. The difference between physicians and GPT-5 did not reach statistical significance (p = 0.100). For theoretical questions, GPT-4 achieved the highest score (85.5%), but statistical significance was not achieved after Bonferroni correction (p = 0.044). For clinical questions, GPT-5 achieved the highest score (77.6%) compared to physicians (70.7%) and GPT-4 (69.0%), although the differences did not reach statistical significance.

Conclusions

There were no statistically significant differences in accuracy detected between GPT-4, GPT-5, and physicians. These results suggest a possible role for LLMs as educational support tools, although confirmation in real-world clinical settings remains necessary. GPT-4 obtained numerically higher accuracy on theoretical questions, whereas GPT-5 scored higher on clinical scenario-based items; however, these differences were not statistically significant. This pattern may indicate evolving performance characteristics across newer generations of LLMs, but such an interpretation remains tentative and requires further validation. Overall, the findings point to the potential usefulness of LLMs in medical education and knowledge-support contexts; however, they should not be interpreted as evidence of clinical competence. Further studies across multiple specialties and real-world healthcare environments are needed to determine their practical applicability.

## Introduction

Recently, artificial intelligence (AI) has become one of the most dynamically developing fields in biomedical sciences. Of particular interest to the medical community are large language models (LLMs), which, thanks to training on billions of words and documents, achieve a high capacity for understanding and generating natural language [1]. These models, such as GPT-4 and the latest GPT-5, can be used in medical education, literature summarization, and supporting knowledge retrieval processes [2,3].

An increasing number of studies indicate that GPT models are capable of answering exam questions with accuracy rates that do not statistically differ from, and sometimes numerically exceed, those of physicians [4,5]. However, there is ongoing controversy regarding the extent to which these models excel in theoretical knowledge (e.g., multiple-choice questions in basic sciences) versus clinical applications, which require contextual reasoning and patient data interpretation [6,7]. Previous analyses have shown that GPT-4 significantly outperforms earlier models (e.g., GPT-3.5) in medical knowledge tests [5,8], and its performance sometimes shows no statistically significant difference from the average achieved by physicians [3].

Nonetheless, there remains a lack of studies directly comparing GPT-5, the newest and more advanced model, with previous model generations and practicing physicians, particularly with respect to theoretical knowledge versus clinical reasoning tasks. Therefore, the primary objective of this study was to compare the performance of physicians, GPT-4, and GPT-5 on questions from the Polish National Cardiology Specialization Examination. The secondary objective was to evaluate differences in performance according to question type (theoretical vs. clinical) and model generation.

## Materials and methods

This comparative observational study was conducted between August 8 and August 15, 2025. The analyzed material consisted of 120 multiple-choice questions derived from the Polish National Cardiology Specialization Examination administered in spring 2025 by the Medical Examination Center (CEM) in Łódź [9]. The official examination questions and corresponding answer key were publicly available on the CEM website.

Each question was independently reviewed and classified into one of two predefined categories based on its primary cognitive requirement: (1) theoretical questions (n = 62), assessing factual knowledge, pathophysiology, pharmacology, and guideline-based recommendations; (2) clinical questions (n = 58), requiring interpretation of clinical scenarios, integration of patient data, diagnostic reasoning, or selection of optimal management strategies.

All questions were included in the analysis. In cases where wording could be considered potentially ambiguous, classification into a theoretical or clinical category was performed independently by the authors, with disagreements resolved by consensus.

The physician group consisted of 153 candidates who completed cardiology specialization training and participated in the spring 2025 examination. Aggregate performance data for this group were obtained from official examination results. All physicians answered the same set of 120 questions under standardized examination conditions defined by the CEM.

Two LLMs, ChatGPT-4 and ChatGPT-5, were evaluated. Each model was tested independently using the same set of exam questions. The evaluations were conducted between August 8 and August 15, 2025, using publicly available versions of both models accessible at that time through the standard user interface. No additional system prompts or task-specific instructions were provided beyond the original examination questions, and all inputs were entered verbatim in Polish. Default model parameters were used, including temperature and sampling settings, as configured by the platform, without manual adjustment. The models were run without access to external databases, web browsing, plugins, or clinical decision-support tools. Each question was submitted in a separate session to minimize potential context carryover. For each question, only a single final answer option was generated and recorded, corresponding to the model’s first response, and no answer refinement or repetition was allowed. This single-response approach was chosen to reflect a real-world user interaction scenario consistent with prior studies evaluating LLMs on medical examinations.

The primary outcome was the percentage of correctly answered questions for each group (physicians, ChatGPT-4, and ChatGPT-5), calculated overall and separately for theoretical and clinical questions. Secondary outcomes included question-level correctness comparisons between groups.

Statistical analysis

Descriptive statistics were used to summarize accuracy rates, presented as percentages with 95% confidence intervals (CI). Because physician performance was provided as an aggregated group average, one-sample proportion Z-tests were utilized to compare the deterministic accuracy of each AI model against the physician cohort baseline. To evaluate differences in performance directly between ChatGPT-4 and ChatGPT-5 on the paired categorical outcomes (correct/incorrect), McNemar’s test was employed. To control for the family-wise error rate across multiple comparisons, a Bonferroni correction was applied, setting the threshold for statistical significance at p < 0.0167. Furthermore, 95% CIs for the differences in proportions between groups were calculated to estimate effect sizes. All analyses were performed using SPSS Statistics software (IBM Corp., Armonk, NY, USA).

## Results

Overall performance

Table 1 presents a comparison of the performance of physicians and AI models on the cardiology exam questions (percentage of correct answers and 95% CI).

**Table 1:** Comparison of the overall performance of physicians and AI models (120 questions). A p-value <0.0167 is considered significant after Bonferroni correction. AI = artificial intelligence; CI = confidence interval

Group	% Correct (95% CI)	Difference vs. physicians (95% CI)	Z-value	P-value (Z-test)
Physicians (n = 153)	72.5% (63.9–81.1)	–	–	–
ChatGPT-4	77.5% (69.3–85.7)	+5.0% (-2.5% to 12.5%)	1.23	0.220
ChatGPT-5	79.2% (71.2–87.2)	+6.7% (-0.6% to 14.0%)	1.64	0.100

**Figure 1:**
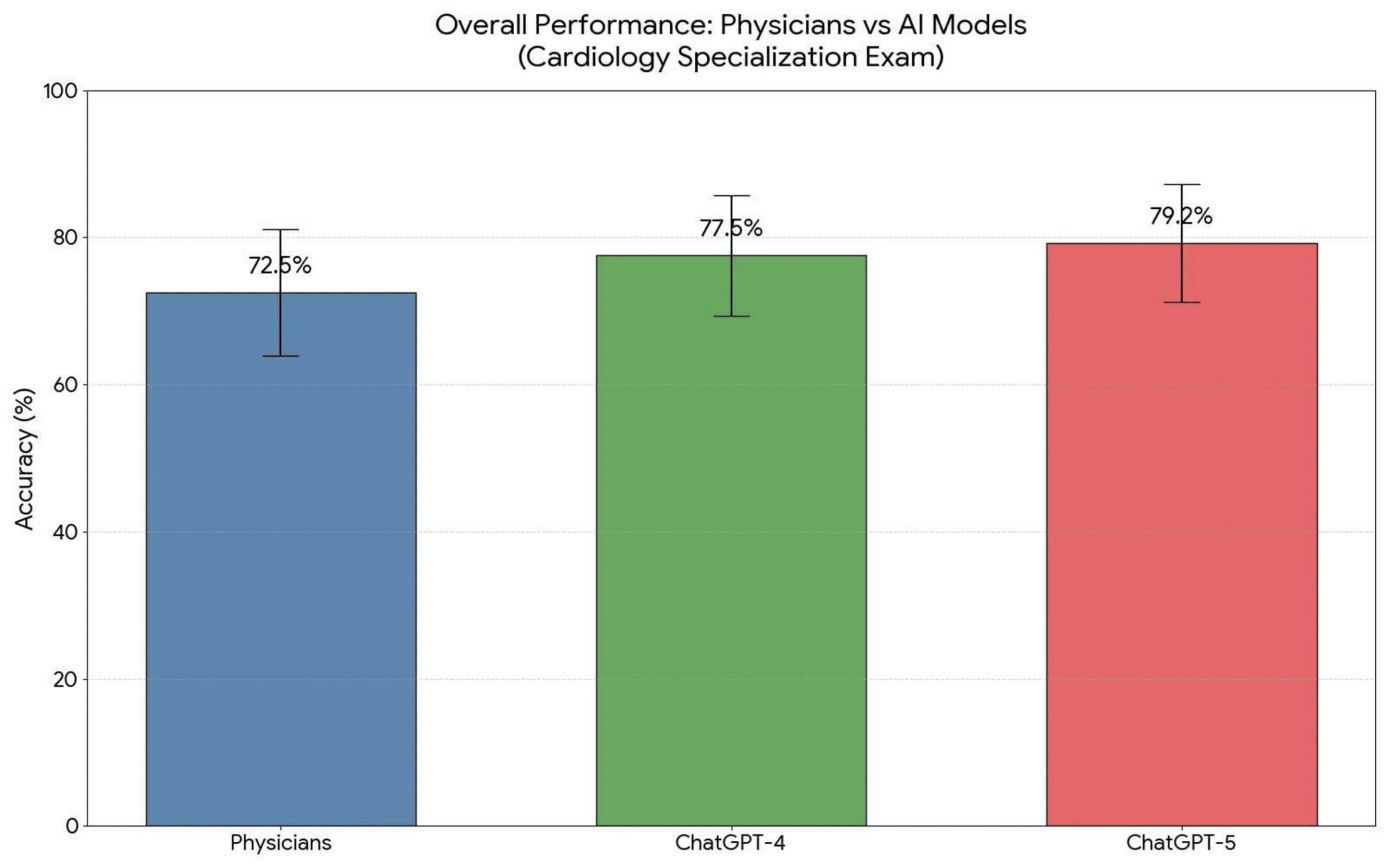
Overall performance comparison of physicians (n = 153), ChatGPT-4, and ChatGPT-5 on the National Specialization Examination in Cardiology. The bars represent the mean percentage of correct answers, while the error bars indicate the 95% CIs. Both AI models achieved scores higher than the physicians' average, with ChatGPT-5 reaching the highest accuracy of 79.2%. AI = artificial intelligence; CI = confidence interval

Physicians, as well as ChatGPT-4 and ChatGPT-5, all achieved the passing threshold of 60%. Both models scored higher than the average performance of physicians (Figure 1). The differences between physicians and ChatGPT-4 were not statistically significant (Z = 1.23, p = 0.220). The differences between physicians and ChatGPT-5 were not statistically significant (Z = 1.64, p = 0.100).

Theoretical questions

Table 2 presents a comparison of the performance of physicians and AI models in cardiology exam questions, focusing on theoretical, general knowledge-based content.

**Table 2:** Comparison of performance in theoretical questions (n = 62). A p-value <0.0167 is considered significant after Bonferroni correction. CI = confidence interval

Group	% Correct (95% CI)	Difference vs. physicians (95% CI)	Z-value	P-value (Z-test)
Physicians (n = 153)	74.3% (62.8–85.8)	–	–	–
ChatGPT-4	85.5% (76.1–94.9)	+11.2% (+2.4% to +20.0%)	2.02	0.044
ChatGPT-5	80.6% (69.9–91.3)	+6.3% (-3.5% to +16.1%)	1.14	0.256

The numerical advantage of ChatGPT-4 over physicians was not statistically significant after Bonferroni correction (Z = 2.02, p = 0.044), while the differences between physicians and ChatGPT-5 were also not statistically significant (Z = 1.14, p = 0.256).

Clinical questions

Table 3 shows the comparison of the performance of physicians and AI models in cardiology exam questions, focusing on clinical aspects.

**Table 3:** Comparison of performance in clinical questions (n = 58). A p-value <0.0167 is considered significant after Bonferroni correction. CI = confidence interval

Group	% Correct (95% CI)	Difference vs. physicians (95% CI)	Z-value	P-value (Z-test)
Physicians (n = 153)	70.7% (58.3–83.1)	–	–	–
ChatGPT-4	69.0% (56.4–81.6)	-1.7% (-13.6% to +10.2%)	-0.29	0.776
ChatGPT-5	77.6% (66.0–89.2)	+6.9% (-3.8% to +17.6%)	1.16	0.248

No significant differences were found between physicians and ChatGPT-4 (Z = -0.29, p = 0.776). The numerical advantage of ChatGPT-5 over physicians did not reach the threshold of statistical significance (Z = 1.16, p = 0.248).

Comparison between ChatGPT-4 and ChatGPT-5

**Figure 2:**
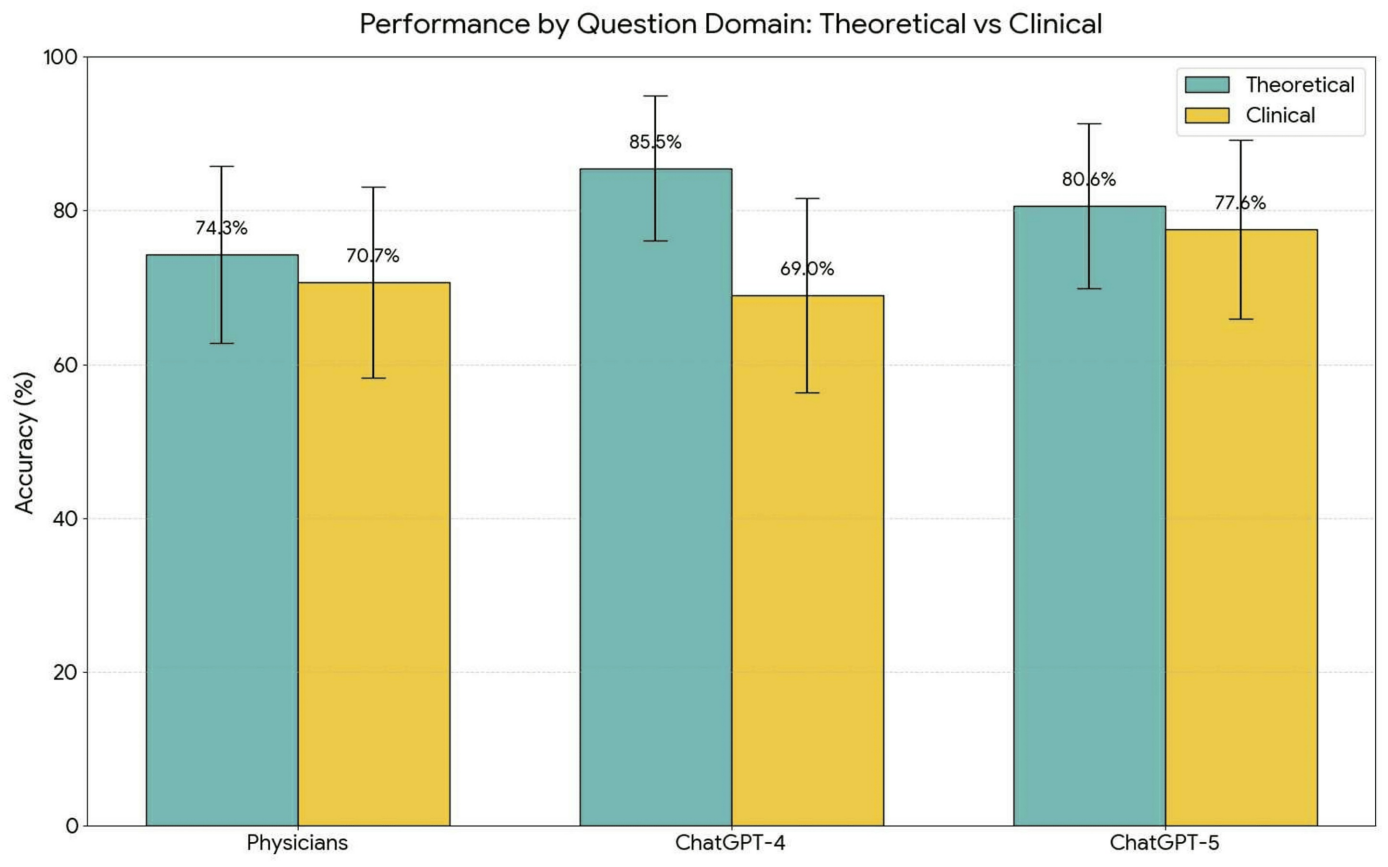
Performance comparison stratified by question domain: theoretical (n = 62) versus clinical (n = 58). ChatGPT-4 demonstrated better performance in theoretical questions (85.5%). In contrast, ChatGPT-5 showed advantage in clinical reasoning questions (77.6%), achieved better score then both physicians and the previous model generation (GPT-4).

In theoretical questions, GPT-4 (85.5%) performed better numerically compared to GPT-5 (80.6%) (Figure 2), although the difference was not statistically significant (Z = -0.73, p = 0.465). In clinical questions, GPT-5 (77.6%) achieved a higher numerical score than GPT-4 (69.0%) (Figure 2), also failing to reach statistical significance (Z = 1.05, p = 0.294) (Table 4).

**Table 4:** Comparison of ChatGPT-4 and ChatGPT-5 performance in theoretical and clinical questions. The difference represents ChatGPT-5’s score relative to ChatGPT-4. A p-value <0.0167 is considered significant after Bonferroni correction. CI = confidence interval

Question type	ChatGPT-4 % (95% CI)	ChatGPT-5 % (95% CI)	Difference (95% CI)	Z-value	P-value (Z-test)
Theoretical (n = 62)	85.5% (76.1–94.9)	80.6% (69.9–91.3)	-4.9% (-18.0% to +8.2%)	-0.73	0.465
Clinical (n = 58)	69.0% (56.4–81.6)	77.6% (66.0–89.2)	+8.6% (-7.5% to +24.7%)	1.05	0.294

Although the differences did not reach statistical significance, they revealed distinct performance profiles of the models, with GPT-4 excelling in theory and GPT-5 in clinical reasoning.

Table 5 presents a summary of exam question numbers, correct answers according to CEM, and answers generated by the ChatGPT-4o and ChatGPT-5 models, as well as information on the type and difficulty of questions and the percentage of correct answers among candidates.

**Table 5:** Summary of exam question numbers along with correct answers based on the CEM and answers generated by the ChatGPT-4o and ChatGPT-5 models. Also included are the question type (clinical or theoretical), the question difficulty rating based on the ChatGPT-4o and ChatGPT-5 models, and the CEM difficulty index (on a scale of 1–5). Additionally, the percentage of examinees who answered each question correctly is presented. Question difficulty index translated into 1-5: 5 = very difficult; 4 = difficult; 3 = moderately difficult; 2 = easy; 1 = very easy. Difficulty index: 0.00-0.19 -> 5 = very difficult; 0.20-0.49 -> 4 = difficult; 0.50-0.69 -> 3 = moderately difficult; 0.70-0.89 -> 2 = easy; 0.90-1.00 -> 1 = very easy. CEM = Medical Examination Center

Question number	Correct answer according to CEM	Correct answer according to ChatGPT 4.0	Correct answer according to ChatGPT 5	Question type 1-clinical, 0-theoretical	Question difficulty according to ChatGPT 4.0	Question difficulty according to ChatGPT 5	How many % of those taking the exam gave the correct answer	Question difficulty index according to CEM translated into 1-5	Question difficulty index according to CEM
1	D	D	D	0	3	3	93.2	2	0.847
2	A	A	A	0	2	2	83.5	2	0.764
3	D	D	D	0	2	1	97.0	1	0.931
4	C	C	C	0	3	3	91.7	2	0.833
5	D	D	D	0	3	4	89.5	2	0.833
6	D	D	D	0	1	2	92.5	1	0.917
7	A	A	A	0	2	3	97.0	1	0.917
8	B	B	B	0	2	2	82.0	2	0.750
9	A	A	B	0	2	2	54.5	3	0.556
10	E	E	B	1	2	2	90.2	2	0.833
11	D	B	B	1	2	2	57.9	3	0.528
12	B	C	B	1	3	2	47.4	4	0.458
13	C	A	A	1	1	1	55.3	3	0.514
14	D	C	A	1	3	3	41.2	4	0.444
15	B	C	C	1	3	3	55.6	3	0.556
16	A	A	A	0	2	2	77.6	2	0.708
17	B	B	B	0	4	4	84.2	2	0.764
18	A	A	A	0	4	3	72.9	4	0.208
19	A	A	A	1	2	2	28.40	4	0.333
20	A	A	A	0	2	3	72.4	3	0.611
21	C	C	C	1	3	4	78.4	3	0.694
22	E	E	E	1	3	2	92.5	2	0.861
23	B	B	B	0	4	3	94.8	1	0.917
24	C	D	D	0	5	4	71.1	3	0.597
25	C	A	C	1	3	3	37.8	4	0.389
26	C	C	B	0	4	4	77.8	3	0.681
27	B	C	B	1	4	4	62.2	3	0.556
28	C	C	C	1	4	4	80.0	2	0.778
29	D	D	D	1	4	3	91.9	2	0.833
30	D	D	D	0	4	4	70.4	3	0.639
31	B	B	B	0	4	4	86.6	2	0.792
32	B	B	B	1	4	4	56.3	4	0.472
33	C	C	C	0	4	4	75.6	2	0.764
34	D	D	D	0	4	3	85.2	2	0.861
35	E	E	E	0	3	3	92.5	2	0.861
36	E	E	E	1	3	4	89.6	2	0.847
37	D	D	D	0	4	4	87.3	2	0.764
38	B	B	B	0	3	2	70.1	2	0.708
39	E	E	E	0	3	3	85.2	2	0.792
40	D	D	D	1	4	2	80.7	2	0.736
41	E	E	E	0	4	3	88.9	4	0.222
42	C	C	C	0	3	2	87.4	2	0.861
43	D	D	D	1	3	2	87.3	2	0.819
44	C	B	B	1	3	3	38.5	4	0.403
45	D	E	C	1	3	3	80.7	2	0.806
46	E	E	E	1	4	4	68.1	3	0.667
47	C	C	C	1	4	4	76.7	2	0.792
48	A	C	B	1	3	3	33.3	4	0.333
49	D	D	D	1	3	2	58.5	3	0.639
50	A	E	E	0	3	4	85.8	2	0.806
51	B	A	B	1	4	3	72.6	2	0.722
52	E	B	B	1	3	3	42.2	4	0.417
53	D	D	D	1	2	2	71.1	3	0.681
54	B	B	B	0	2	2	42.2	4	0.431
55	C	C	C	1	2	3	74.1	2	0.722
56	B	B	B	1	3	4	90.4	2	0.833
57	E	E	E	1	3	3	39.3	4	0.458
58	B	B	B	0	3	3	58.5	4	0.444
59	D	D	D	1	4	4	25.20	4	0.333
60	D	B	B	0	4	4	32.6	4	0.347
61	B	B	B	1	3	2	81.3	2	0.778
62	D	C	D	1	4	4	60.2	3	0.583
63	E	E	E	0	3	2	89.6	2	0.861
64	D	D	D	0	4	3	83.0	2	0.750
65	B	A	A	1	4	4	75.6	2	0.722
66	D	D	D	0	2	2	42.2	2	0.722
67	C	C	C	0	3	3	82.2	2	0.708
68	D	C	C	0	3	3	67.4	3	0.625
69	C	C	C	1	3	3	95.6	1	0.944
70	B	A	B	1	4	4	59.3	3	0.556
71	D	A	E	1	2	2	68.9	3	0.500
72	E	E	E	0	3	3	76.3	3	0.694
73	D	D	D	0	4	4	84.4	2	0.833
74	E	E	E	0	4	4	70.4	2	0.736
75	B	B	B	0	4	3	63.0	3	0.611
76	C	C	C	1	4	4	93.3	1	0.903
77	B	B	B	0	4	3	89.6	2	0.819
78	D	D	D	0	3	2	85.9	2	0.847
79	C	C	C	1	4	4	84.4	2	0.875
80	B	C	C	1	4	4	60.7	3	0.569
81	C	A	E	0	3	4	78.5	2	0.750
82	C	C	C	0	4	4	86.6	2	0.764
83	D	D	D	0	3	3	65.2	3	0.625
84	B	B	A	0	3	3	45.5	4	0.389
85	E	A	D	0	3	4	26.90	4	0.306
86	A	A	A	1	2	2	78.5	2	0.778
87	B	B	B	0	3	3	54.5	4	0.472
88	B	B	B	0	3	3	48.5	4	0.486
89	A	C	D	0	3	3	10.400	5	0.125
90	A	C	C	0	4	4	86.7	4	0.417
91	B	B	B	1	4	4	55.6	3	0.583
92	A	A	A	0	3	3	86.7	2	0.792
93	D	D	D	0	3	3	67.4	3	0.625
94	C	C	C	1	3	2	36.3	2	0.361
95	D	D	D	1	3	3	94.1	1	0.903
96	C	C	C	1	3	3	70.4	3	0.681
97	B	B	B	1	3	4	78.4	2	0.708
98	E	E	E	0	2	3	50.4	3	0.528
99	E	E	E	0	2	2	61.9	3	0.583
100	C	C	C	1	3	3	82.2	2	0.764
101	D	D	D	1	3	3	83.0	2	0.736
102	C	C	C	1	2	3	71.1	3	0.639
103	A	A	A	1	2	2	97.8	1	0.958
104	B	C	B	1	3	3	90.4	2	0.806
105	C	C	C	0	2	2	83.0	2	0.764
106	C	C	C	1	2	2	88.9	2	0.833
107	D	D	D	1	3	3	97.0	1	0.944
108	A	A	A	1	3	3	87.4	2	0.806
109	E	A	A	0	3	3	66.4	3	0.625
110	D	D	D	0	3	3	68.1	3	0.681
111	C	C	C	0	2	2	81.3	2	0.833
112	C	C	C	1	3	3	75.6	3	0.681
113	E	E	B	1	3	3	71.9	2	0.736
114	D	D	D	1	2	3	94.1	2	0.875
115	A	A	A	0	3	3	91.9	2	0.889
116	C	C	C	0	3	3	87.4	2	0.819
117	C	C	C	1	3	3	83.7	2	0.792
118	B	B	B	0	3	2	58.5	3	0.542
119	C	C	C	1	2	2	81.5	2	0.847
120	E	E	E	0	2	2	84.4	2	0.792

## Discussion

In this study, we compared the performance of physicians and two generations of LLMs, ChatGPT-4 and ChatGPT-5, on exam questions from the National Cardiology Specialty Examination. The analysis included both theoretical and clinical questions, allowing us to assess differences based on the type of knowledge required to answer correctly.

The results indicate that there were no statistically significant differences in accuracy between the AI models and physicians, with numerically higher scores observed for the AI in several comparisons. Overall, ChatGPT-5 achieved the highest percentage of correct answers (79.2%) compared to both ChatGPT-4 (77.5%) and physicians (72.5%). However, the difference between physicians and GPT-5 was not statistically significant (Z = 1.64, p = 0.100). Categorizing the questions revealed differences in performance profiles between the models and physicians. For theoretical questions, ChatGPT-4 achieved the best results (85.5%). However, for clinical questions, ChatGPT-5 achieved better results (77.6%) than physicians (70.7%) and GPT-4 (69.0%), although it did not reach statistical significance.

These results are consistent with a broader trend observed in the global literature, confirming the high effectiveness of AI in medical examinations. Similar or comparable results have been reported in recent studies of other specialties in Poland. Latkowska et al. reported that the ChatGPT-5 model achieved a score of 76.47% in the endocrinology exam, a result very similar to that obtained in our cardiology study [10]. Similarly, in vascular surgery, the GPT-4o model achieved a score of 73.3%, which is consistent with our results [11]. However, it is worth noting that in some areas, such as gynecology and obstetrics and pediatric surgery, AI models (specifically, Gemini 2.5 Pro) achieved even higher scores, exceeding 96% and 85%, respectively [12,13]. These discrepancies may be due to the specificity of the questions in each area; cardiology often requires complex interpretation of multi-parameter clinical data, which can be more challenging than questions based primarily on factual knowledge.

An interesting observation from this study is the difference in response profiles between the model generations. The GPT-4 model obtained the highest numerical score in theoretical questions (85.5%), confirming its ability to accurately process textbook knowledge. This is consistent with reports from Spain, where the GPT-4 achieved a higher score (81.8%) in the neurology exam than older models [14]. In contrast, the newer model, GPT-5, achieved a better score in clinical questions (77.6% compared to 69.0% for the GPT-4). This may suggest a potential trend toward improved contextual reasoning in newer model generations; however, this interpretation remains speculative and requires further validation. This represents a significant advance compared to previous studies, for example, in radiology, where language models such as Bard and Bing struggled with tasks requiring data integration and case vignettes [15].

Our results are also supported by recent findings in child and adolescent psychiatry, where the GPT-5 achieved a high score of 80.8% [16]. These findings may suggest a possible convergence of performance around the 70-80% range across different specialties; however, this observation requires confirmation in larger studies.

Importantly, examination performance does not directly translate into real-world clinical competence. Clinical decision-making involves additional elements such as patient interaction, procedural skills, ethical judgment, and integration of complex diagnostic data, which were not assessed in this study. Therefore, the results should be interpreted as reflecting knowledge-based reasoning ability rather than readiness for independent clinical practice.

Study limitations

This study has several limitations. First, only questions from a single specialty exam (Cardiology, Spring 2025) were analyzed, which limits the generalizability of the results to other medical specialties. Second, the models were evaluated under exam conditions, which do not fully reflect the complexity of real-world clinical practice, including patient interaction, physical examination, and interpretation of imaging and laboratory results. Furthermore, the focus was solely on the correctness of answers, without assessing the quality or clinical relevance of the models’ reasoning.

Another limitation is that physicians’ performance data were available only in aggregated form, which precluded full pairwise comparisons between physicians and AI models. The number of questions in the subgroups (62 theoretical questions and 58 clinical questions) may have been insufficient to detect moderate differences; therefore, the absence of statistical significance should not be interpreted as equivalence between the models and physicians.

Moreover, the analysis of physicians’ results was based on aggregated data, whereas each AI model generated only one answer per question, introducing a methodological asymmetry and limiting direct comparability. The study also did not include a formal qualitative error analysis, which could have revealed systematic weaknesses or reasoning patterns in both the models and physicians.

It is also important to note that the responses of language models may vary depending on generation parameters and stochastic processes. As each model provided only a single answer per question, the results may not reflect the full variability in performance over multiple attempts. Future studies should consider multiple iterations and probabilistic analysis to obtain more reliable estimates of model accuracy.

## Conclusions

In conclusion, although LLMs are not intended to replace human specialists, their strong performance in the spring 2025 cardiology examination indicates potential usefulness as adjunctive educational and knowledge-support tools. The observed pattern, with GPT-4 performing better on knowledge-based items and GPT-5 achieving higher scores in clinically oriented questions, may suggest ongoing improvements in handling complex information; however, this interpretation remains preliminary and requires further validation. Future research should extend beyond standardized examination settings and focus on prospective evaluation in real-world clinical environments. Such studies are necessary to assess safety, reliability, and practical relevance, and to better define the appropriate scope and limitations of LLM use in healthcare contexts.

## Appendices

Exam questions

Question 1 - PES Cardiology, Spring Session 2025

Indicate the correct recommendations concerning a patient resuscitated after out-of-hospital cardiac arrest (OHCA) according to the 2023 ESC/PTK guidelines on acute coronary syndromes (ACS):

1. a primary PCI strategy is recommended in patients resuscitated after OHCA with persistent ST-segment elevation on ECG;

2. routine, immediate coronary angiography should be considered after resuscitated OHCA in hemodynamically stable patients without ST-segment elevation;

3. routine, immediate coronary angiography is not recommended after resuscitated OHCA in hemodynamically stable patients without ST-segment elevation; neurological prognostication (not earlier than 72 hours after hospital admission) is recommended in all unconscious patients after resuscitated OHCA;

4. neurological prognostication (not later than 72 hours after hospital admission) is recommended in all unconscious patients after resuscitated OHCA.

The correct answer is:

A. 1, 2, 4;

B. 1, 2, 5;

C. 3, 5;

D. 1, 3, 4;

E. 2, 3, 5.

Correct answer: D

Question 2 - PES Cardiology, Spring Session 2025

The default treatment strategy after ACS treated with PCI + DES in a patient requiring NOAC therapy due to concomitant atrial fibrillation includes:

A. triple therapy (two antiplatelet agents + NOAC) for one week, followed by dual therapy (antiplatelet agent - preferably a P2Y12 inhibitor + NOAC) up to 12 months after PCI, and subsequently NOAC monotherapy;

B. triple therapy (two antiplatelet agents + NOAC) for one week, followed by dual therapy (antiplatelet agent - preferably ASA + NOAC) up to 12 months after PCI, and subsequently NOAC monotherapy;

C. triple therapy (two antiplatelet agents + NOAC) for one month, followed by dual therapy (antiplatelet agent - preferably a P2Y12 inhibitor + NOAC) up to 12 months after PCI, and subsequently NOAC monotherapy;

D. triple therapy (two antiplatelet agents + NOAC) for one month, followed by dual therapy (antiplatelet agent - preferably ASA + NOAC) up to 12 months after PCI, and subsequently NOAC monotherapy;

E. answers A and B are correct.

Correct answer: A

Question 3 - PES Cardiology, Spring Session 2025

Which of the following is not a method of atherosclerotic plaque modification during PCI?

A. orbital atherectomy;

B. cutting balloon;

C. intravascular lithotripsy (IVL);

D. fractional flow reserve (FFR);

E. rotational atherectomy (rotablation).

Correct answer: D

Question 4 - PES Cardiology, Spring Session 2025

Indicate the incorrect recommendations regarding cardiogenic shock in the course of ACS:

1. in patients with ACS complicated by shock, immediate coronary angiography and PCI of the infarct-related artery are recommended;

2. emergency CABG is recommended in ACS-related shock if PCI of the infarct-related artery is not feasible or unsuccessful;

3. in the case of hemodynamic instability, urgent repair of mechanical complications of ACS is recommended according to Heart Team decision;

4. in the case of hemodynamic instability, urgent repair of mechanical complications of ACS is not recommended due to poor long-term prognosis;

5. routine use of intra-aortic balloon pump (IABP) is not recommended in ACS with shock without mechanical complications;

6. IABP should be considered in ACS with shock without mechanical complications.

The correct answer is:

A. 2, 3, 4, 5;

B. 1, 3, 4, 6;

C. 4, 6;

D. 2, 5;

E. 1, 6.

Correct answer: C

Question 5 - PES Cardiology, Spring Session 2025

Indicate the false recommendations concerning bradyarrhythmias in ACS patients with sinus bradycardia and poor hemodynamic tolerance or high-grade AV block without a stable escape rhythm:

1. use of positively chronotropic drugs (adrenaline, vasopressin and/or atropine) is recommended;

2. temporary pacing is recommended in the absence of response to atropine;

3. urgent coronary angiography and PCI are recommended if the patient has not previously undergone reperfusion therapy;

4. if there is a response to atropine, coronary angiography should be postponed for 48 hours;

5. permanent pacing should be considered even if high-risk AV block resolves after revascularization due to high risk of recurrence;

6. permanent pacing is not recommended if AV block resolves after revascularization or spontaneously.

The correct answer is:

A. 1, 3, 6;

B. 1, 2, 3, 4, 6;

C. 1, 3, 5;

D. 4, 5;

E. 4, 6.

Correct answer: D

Question 6 - PES Cardiology, Spring Session 2025

After 12 months following ACS, which of the following cannot be used for long-term antiplatelet therapy?

A. acetylsalicylic acid;

B. clopidogrel;

C. ticagrelor;

D. fondaparinux;

E. prasugrel.

Correct answer: D

Question 7 - PES Cardiology, Spring Session 2025

Indicate the correct recommendations concerning initial management of ACS patients:

1. recording additional ECG leads (V3R, V4R and V7-V9) is recommended in inferior STEMI or when complete vessel occlusion is suspected and standard leads are inconclusive;

2. serial hs-cTn algorithms (0 h/1 h or 0 h/2 h) are recommended to confirm or rule out NSTEMI;

3. serial hs-cTn algorithms (0 h/3 h or 0 h/6 h) are recommended to confirm or rule out NSTEMI;

4. an additional test after 3 h is recommended if the first two hs-cTn measurements in the 0 h/1 h algorithm are inconclusive and no alternative diagnosis explains the patient’s condition;

5. an additional test after 6 h is recommended if the first two hs-cTn measurements in the 0 h/3 h algorithm are inconclusive and no alternative diagnosis explains the patient’s condition;

6. prasugrel should be considered preferentially over ticagrelor in ACS patients qualified for PCI;

7. in NSTE-ACS patients not scheduled for early coronary angiography (within 24 h), fondaparinux is recommended.

The correct answer is:

A. 1, 2, 4, 6, 7;

B. 1, 3, 5, 6;

C. 1, 3, 5, 7;

D. 2, 3, 7;

E. 2, 5, 6.

Correct answer: A

Question 8 - PES Cardiology, Spring Session 2025

Intravascular imaging modalities used during PCI and in diagnosing causes of ACS without significant coronary stenosis include:

1. intravascular ultrasound (IVUS);

2. fractional flow reserve (FFR);

3. optical coherence tomography (OCT);

4. non-hyperemic indices (RFR, DPR, iwFR).

The correct answer is:

A. 1, 2;

B. 1, 3;

C. 1, 4;

D. 2, 4;

E. 2, 3.

Correct answer: B

Question 9 - PES Cardiology, Spring Session 2025

In selected cases, DAPT duration may be shortened. However, shortening below which duration is not recommended after ACS in patients not receiving additional anticoagulation for other indications?

A. 30 days;

B. 60 days;

C. 3 months;

D. 6 months;

E. 12 months;

Correct answer: A

Question 10 - PES Cardiology, Spring Session 2025

A 69-year-old man with no prior treatment history was admitted with NSTEMI. Coronary angiography revealed diffuse coronary disease, and the patient was referred for CABG. Post-CABG laboratory values: hemoglobin 12.6 g/dL, creatinine 0.8 mg/dL, no active bleeding, no arrhythmias. Which antithrombotic/antiplatelet therapy should be prescribed at discharge?

A. ASA 150 mg once daily;

B. ASA 75 mg once daily;

C. rivaroxaban 20 mg once daily;

D. clopidogrel 75 mg once daily;

E. ASA 75 mg once daily + clopidogrel 75 mg once daily for 12 months, then de-escalation to single antiplatelet therapy.

Correct answer: E

Question 11 - PES Cardiology, Spring Session 2025

A 70-year-old man experienced a brief first-ever syncope at home during micturition, without prodromal symptoms, resulting in head trauma. Physical examination was unremarkable. Blood pressure supine: 120/70 mmHg; after 3 minutes standing: 110/75 mmHg. ECG shown below. The most likely cause of syncope is:

A. orthostatic;

B. situational;

C. reflex;

D. cardiac;

E. psychogenic;

Correct answer: D

Question 12 - PES Cardiology, Spring Session 2025

A 78-year-old patient on chronic hemodialysis was brought to the emergency department after syncope. Currently asymptomatic. ECG shown below, paper speed 25 mm/s. Which intervention will most likely be required?

A. potassium supplementation;

B. intravenous calcium administration;

C. intravenous magnesium administration;

D. coronary angiography;

E. amiodarone administration.

Correct answer: B

Question 13 - PES Cardiology, Spring Session 2025

A 67-year-old woman complains of dizziness upon standing. Resting BP supine: 100/60 mmHg; after standing: 88/58 mmHg. Indicate the correct statement:

A. systolic BP dropped by 12 mmHg, and the threshold for diagnosing orthostatic hypotension (OH) is 20 mmHg, therefore OH is not diagnosed;

B. diastolic BP decreased, which is sufficient to diagnose OH;

C. the patient has orthostatic hypotension;

D. lack of pulse data prevents assessment of OH;

E. SBP drop below 85 mmHg is necessary to diagnose OH.

Correct answer: C

Question 14 - PES Cardiology, Spring Session 2025

A 62-year-old woman, 6 months after anterior-lateral STEMI treated with PCI of the LAD with DES, presents with a 24-hour Holter ECG showing 910 isolated monomorphic PVCs and five episodes of non-sustained VT (3-7 beats). She reports no angina, dyspnea, palpitations or syncope and is on optimal medical therapy. TTE shows LVEF = 40%. What should the cardiologist recommend?

A. further observation and repeat TTE in 3-6 months;

B. initiation of amiodarone;

C. implantation of an ICD;

D. invasive electrophysiological study (EPS);

E. ablation of ventricular ectopic focus.

Correct answer: D

Question 15 - PES Cardiology, Spring Session 2025

What should be the further management of a 30-year-old man without structural heart disease, with spontaneous ECG changes consistent with type 1 Brugada syndrome and one syncope of unclear etiology?

A. perform a sodium channel blocker test (e.g. ajmaline);

B. consider implantation of an implantable loop recorder (ILR);

C. recommend ICD implantation;

D. consider quinidine therapy;

E. recommend further observation;

Correct answer: B

Question 16 - PES Cardiology, Spring Session 2025

In a patient with prolonged QT and recurrent polymorphic ventricular tachycardia, which treatment must not be used?

A. amiodarone;

B. transvenous pacing at 120/min;

C. magnesium;

D. isoproterenol;

E. answers B and C are correct.

Correct answer: A

Question 17 - PES Cardiology, Spring Session 2025

In patients with hypertrophic cardiomyopathy (HCM) and atrial fibrillation (AF):

1. rate control is preferred;

2. DOAC therapy should be initiated according to CHA₂DS₂-VASc score;

3. anticoagulation is recommended in all patients with HCM and AF or atrial flutter in the absence of contraindications;

4. AF ablation is recommended for rhythm control after failure or intolerance of pharmacotherapy in paroxysmal or persistent AF;

5. lifestyle modification is recommended to reduce AF occurrence.

The correct answer is:

A. 1, 2, 5;

B. 3, 4, 5;

C. 1, 3, 5;

D. only 3;

E. 1, 5.

Correct answer: B

Question 18 - PES Cardiology, Spring Session 2025

A patient with permanent AF after recent implantation of a bioprosthetic mitral valve should be discharged on which anticoagulant regimen?

A. VKA alone for 3 months, then possible switch to NOAC;

B. DAPT: ASA + clopidogrel;

C. VKA + ASA;

D. NOAC + ASA;

E. NOAC or VKA.

Correct answer: A

Question 19 - PES Cardiology, Spring Session 2025

A 24-year-old agitated man was brought to the ED after using cocaine 3 hours earlier. BP 180/120 mmHg, SpO₂ 97%. ECG shown below, paper speed 25 mm/s. Which intervention will most likely be required?

A. diazepam administration;

B. metoprolol administration;

C. oxygen therapy;

D. gastric lavage;

E. electrical cardioversion.

Correct answer: A

Question 20 - PES Cardiology, Spring Session 2025

Patient-related factors increasing thromboembolic risk that affect the target INR in mechanical heart valves include all of the following except:

A. presence of a prosthesis in the aortic position;

B. prior thromboembolic event;

C. mitral stenosis of any degree;

D. atrial fibrillation;

E. LVEF <35%.

Question 21 - PES Cardiology, Spring Session 2025

A 66-year-old woman reports episodes of palpitations. History includes type 2 diabetes and hypertension. Holter ECG reveals arrhythmia episodes as shown below (paper speed 25 mm/s). Indicate the correct statement:

A. DOAC should be prescribed because AF is present on Holter ECG;

B. ASA should be prescribed because AF is present and the patient is >65 years old;

C. no AF episode qualifying for anticoagulation was detected on ECG;

D. regardless of ECG findings, DOAC is indicated due to diabetes and hypertension;

E. regardless of ECG findings, DOAC will be indicated after age 65 due to diabetes and hypertension.

Correct answer: C

Question 22 - PES Cardiology, Spring Session 2025

A 72-year-old man with diabetes, hypertension and peripheral arterial disease underwent pulmonary vein isolation for AF. After 3 months, he remains arrhythmia-free. What should be the recommendation regarding anticoagulation (DOAC)?

A. no anticoagulation required;

B. prescribe ASA;

C. monitor heart rate and restart DOAC if tachycardia occurs;

D. monitor heart rate, perform ECG if tachycardia occurs, and restart DOAC if AF is confirmed;

E. lifelong DOAC therapy.

Correct answer: E

Question 23 - PES Cardiology, Spring Session 2025

In a patient treated with verapamil, which DOAC requires dose reduction due to increased bleeding risk?

A. apixaban;

B. dabigatran;

C. edoxaban;

D. rivaroxaban;

E. none of the above.

Correct answer: B

Question 24 - PES Cardiology, Spring Session 2025

Indicate the false statements regarding antiplatelet/anticoagulant therapy:

1. rivaroxaban 2×2.5 mg may be considered as an alternative to ASA in secondary prevention of ASCVD;

2. clopidogrel 75 mg once daily may be preferred over ASA in patients with established ASCVD;

3. concomitant use of proton pump inhibitors may be considered in patients receiving antiplatelet therapy with high gastrointestinal bleeding risk;

4. in patients with diabetes or very high cardiovascular risk, low-dose ASA may be considered for primary prevention;

5. antiplatelet therapy should be considered in individuals with low/moderate CV risk for primary prevention, preferring clopidogrel in those at increased bleeding risk.

The correct answer is:

A. 1, 3, 4;

B. 2, 3;

C. 1, 3, 5;

D. 2, 5;

E. 4, 5.

Correct answer: C

Question 25 - PES Cardiology, Spring Session 2025

An 83-year-old man with a mechanical mitral valve implanted 10 years ago, chronically treated with VKA, presents with pneumonia. INR is 12, no bleeding. Apart from temporarily withholding VKA, what should be recommended?

A. 10 mg vitamin K IV slowly;

B. PCC and/or fresh frozen plasma;

C. oral vitamin K 2.5-5 mg;

D. answers A and B;

E. only temporary interruption of VKA.

Correct answer: C

Question 26 - PES Cardiology, Spring Session 2025

LMWH therapy during pregnancy requires anti-Xa monitoring. What is the therapeutic anti-Xa range for a mechanical aortic valve?

A. 0.6-0.8 IU/mL;

B. 0.6-1.0 IU/mL;

C. 0.8-1.2 IU/mL;

D. 1.0-1.2 IU/mL;

E. 1.2-1.5 IU/mL.

Correct answer: C

Question 27 - PES Cardiology, Spring Session 2025

A 58-year-old patient with permanent AF, prior MI, LVEF 40%, and a mechanical Medtronic Hall mitral valve. Recommended INR target:

A. 2.5-3.5;

B. 3.0;

C. 3.0-3.5;

D. 3.5;

E. 4.0.

Correct answer: B

Question 28 - PES Cardiology, Spring Session 2025

A patient with a mechanical aortic valve is scheduled for elective cholecystectomy. Perioperative management should include:

1. temporary VKA interruption to INR <1.5 (warfarin ~5 days, acenocoumarol ~3 days);

2. interruption of both VKAs one week prior;

3. bridging with therapeutic LMWH;

4. post-operative LMWH after 24 h and VKA on day 2;

5. post-operative LMWH after 12-24 h and VKA on day 1.

Correct answer:

A. 1, 3, 4;

B. 2, 3, 4;

C. 1, 3, 5;

D. 2, 3, 5;

E. 2, 4.

Correct answer: C

Question 29 - PES Cardiology, Spring Session 2025

In a patient with Child-Pugh B liver cirrhosis due to chronic alcohol abuse, which is not recommended?

A. apixaban;

B. dabigatran;

C. edoxaban;

D. rivaroxaban;

E. left atrial appendage closure.

Correct answer: D

Question 30 - PES Cardiology, Spring Session 2025

A patient after myocarditis plans to return to sports. Which statements are true?

1. return to high-intensity exercise may be considered after 3-6 months if asymptomatic, with normal biomarkers, normal LVEF, no active inflammation or fibrosis on CMR, good exercise capacity, and no complex ventricular arrhythmias;

2. patients with residual myocardial damage and persistent LV dysfunction may engage in high-intensity sport;

3. myocarditis permanently disqualifies from high-intensity sports;

4. myocarditis carries relapse risk; presence of LGE during acute phase is linked to increased major cardiac events;

5. patients with extensive scar (>20% LGE) and persistent LV dysfunction should refrain from low-intensity sports.

Correct answer:

A. all;

B. 2, 3, 5;

C. 1, 2, 4;

D. 1, 4;

E. 1, 4, 5.

Correct answer: D

Question 31 - PES Cardiology, Spring Session 2025

Indicate false statements regarding AF/AFL management in athletes:

1. in structurally normal hearts with well-tolerated AF, physical activity without antiarrhythmic therapy may be considered;

2. cavotricuspid isthmus ablation should be considered in documented AFL before intensive exercise;

3. contact sports may be considered in AF patients on NOAC but not on VKA;

4. after pill-in-the-pocket flecainide/propafenone, immediate return to intense sports is acceptable;

5. AF ablation is recommended in symptomatic recurrent AF or when athletes refuse pharmacotherapy.

Correct answer:

A. 1, 3;

B. 3, 4;

C. 1, 2, 4;

D. 3, 5;

E. 3, 4, 5.

Correct answer: B

Question 32 - PES Cardiology, Spring Session 2025

A patient with dilated cardiomyopathy (DCM) plans high-intensity sport. Which findings are not absolute contraindications?

1. symptoms, cardiac arrest history, or unexplained syncope;

2. LVEF = 45%;

3. frequent/complex ventricular arrhythmias;

4. extensive LGE 10%;

5. high-risk genotype (LMNA or FLNC mutation).

Correct answer:

A. 1, 2;

B. 2, 4;

C. 4, 5;

D. all are contraindications;

E. none are contraindications.

Correct answer: B

Question 33 - PES Cardiology, Spring Session 2025

Which EPS finding with isoproterenol does not indicate a high-risk accessory pathway?

A. inducible AVRT or AF;

B. AF with pre-excitation and RR ≤250 ms;

C. antegrade refractory period >250 ms;

D. multiple accessory pathways;

E. septal pathway location.

Correct answer: C

Question 34 - PES Cardiology, Spring Session 2025

Indicate false statements regarding exercise in PSVT and pre-excitation:

1. athletes with PSVT should stop exercise during arrhythmia;

2. class I antiarrhythmics have no role due to proarrhythmic risk;

3. ablation should be considered for competitive sports and is equally safe/effective;

4. ablation is recommended in symptomatic pre-excitation;

5. EPS is not recommended in asymptomatic competitive athletes with pre-excitation.

Correct answer:

A. 1, 2, 3;

B. 2, 3, 4;

C. 3, 4, 5;

D. only 5;

E. 4, 5.

Correct answer: D

Question 35 - PES Cardiology, Spring Session 2025

False recommendations regarding sports in patients with PM or ICD:

1. shared decision-making after 3 months without arrhythmias in Brugada ICD patients;

2. sports (except contact) may be considered in PM patients without malignant arrhythmia substrate;

3. consider device/electrode placement adjustments to avoid trauma;

4. Holter and device interrogation during/after exercise is recommended;

5. ICD implantation can replace disease-specific sports restrictions;

6. decisions should consider arrhythmic substrate and shock risk.

Correct answer:

A. only 1;

B. 1, 2;

C. only 3;

D. 3, 4, 6;

E. only 5.

Correct answer: E

Question 36 - PES Cardiology, Spring Session 2025

A 32-year-old woman, 32 weeks pregnant, asymptomatic severe aortic stenosis (AVA 0.95 cm², Vmax 4.2 m/s). Appropriate management:

A. dobutamine stress echo;

B. balloon valvuloplasty;

C. early cesarean section;

D. valve replacement postpartum;

E. observation and follow-up before delivery.

Correct answer: E

Question 37 - PES Cardiology, Spring Session 2025

Incorrect recommendations for AF/SVT management during pregnancy:

1. immediate cardioversion for unstable tachyarrhythmias;

2. IV amiodarone as first-line cardioversion;

3. rhythm control preferred with beta-blockers;

4. NOAC allowed only in 2nd trimester;

5. flecainide/propafenone/sotalol if AV-nodal blockers fail;

6. digoxin/verapamil if beta-blockers ineffective.

Correct answer:

A. only 1;

B. 2, 6;

C. 4, 6;

D. 2, 4;

E. 3, 4, 5.

Correct answer: D

Question 38 - PES Cardiology, Spring Session 2025

Which of the following are associated with a high risk of pre-eclampsia?

1. hypertensive disease during a previous pregnancy;

2. first pregnancy;

3. chronic kidney disease;

4. age >40 years;

5. systemic lupus erythematosus or antiphospholipid syndrome;

6. BMI >35 kg/m² at the first visit;

7. type 1 or type 2 diabetes mellitus;

8. multiple pregnancy;

9. chronic arterial hypertension;

10. interpregnancy interval >10 years.

The correct answer is:

A. 1, 2, 5, 7, 9;

B. 1, 3, 5, 7, 9;

C. 2, 4, 6, 8, 10;

D. 2, 4, 6, 9;

E. 1, 4, 6, 9.

Correct answer: B

Question 39 - PES Cardiology, Spring Session 2025

Indicate the true statements regarding arterial hypertension in pregnancy:

1. women at high or moderate risk of pre-eclampsia should be advised to take ASA 100-150 mg once daily from week 12 to week 36-37 of pregnancy;

2. non-pharmacological treatment of hypertension during pregnancy plays a limited role;

3. the drug of choice in pre-eclampsia associated with pulmonary edema is nitroglycerin;

4. in pre-eclampsia, intravascular volume is increased, therefore treatment should be started with diuretics;

5. preferred drugs for chronic treatment during pregnancy are labetalol, sustained-release nifedipine, and methyldopa;

6. to prevent pre-eclampsia in women with low dietary calcium intake (<600 mg/day), calcium supplementation (1.5-2 g/day orally) is recommended from the first prenatal visit.

The correct answer is:

A. all of the above;

B. 1, 2, 3, 4, 5;

C. 2, 3, 4, 5, 6;

D. 1, 2, 4, 5;

E. 1, 2, 3, 5, 6.

Correct answer: E

Question 40 - PES Cardiology, Spring Session 2025

A 31-year-old woman with a mechanical aortic valve implanted 3 years ago presents to the outpatient clinic stating that she is 6 weeks pregnant. She takes warfarin 6 mg once daily. What antithrombotic treatment would you recommend during pregnancy?

A. continuation of warfarin throughout pregnancy and the peripartum period;

B. continuation of warfarin throughout pregnancy with switching to UFH before delivery;

C. switching warfarin to LMWH until delivery;

D. switching warfarin to LMWH in the first trimester, then resuming oral anticoagulation, with switching to UFH before delivery;

E. use of warfarin during the first two trimesters, then switching to LMWH until delivery.

Correct answer: D

Question 41 - PES Cardiology, Spring Session 2025

Pregnancy should be discouraged and intervention before pregnancy recommended in patients:

1. symptomatic with severe aortic stenosis (AS);

2. asymptomatic with severe AS and LVEF <50%;

3. with bicuspid aortic valve (BAV) and ascending aorta diameter >45 mm (22 mm/m² BSA);

4. with BAV and ascending aorta diameter >50 mm (27 mm/m² BSA);

5. with mitral stenosis and valve area <2 cm².

The correct answer is:

A. 1, 3;

B. 1, 4, 5;

C. 1, 3, 5;

D. 2, 3, 5;

E. 1, 2, 4.

Correct answer: E

Question 42 - PES Cardiology, Spring Session 2025

Mavacamten is:

1. a drug registered in the EU for treatment of symptomatic hypertrophic cardiomyopathy (HCM) with left ventricular outflow tract obstruction (LVOTO);

2. a drug registered in the EU for treatment of symptomatic HCM without LVOTO;

3. a selective, reversible inhibitor of cardiac myosin;

4. a drug registered in the EU for treatment of pulmonary arterial hypertension;

5. a phosphodiesterase type 5 inhibitor.

The correct answer is:

A. only 1;

B. only 2;

C. 1, 3;

D. 3, 4;

E. 4, 5.

Correct answer: C

Question 43 - PES Cardiology, Spring Session 2025

In patients with HFrEF and type 2 diabetes mellitus, which drug should not be used?

A. sotagliflozin;

B. sitagliptin;

C. semaglutide;

D. saxagliptin;

E. metformin;

Correct answer: D

Question 44 - PES Cardiology, Spring Session 2025

Iron supplementation in patients with chronic HF and iron deficiency should be administered:

1. intravenously using ferric carboxymaltose;

2. intravenously using ferric derisomaltose;

3. orally or intravenously with any iron preparation;

4. if transferrin saturation <20% and ferritin <250 ng/mL;

5. if transferrin saturation <30% and ferritin <250 ng/mL.

The correct answer is:

A. only 1;

B. 1, 2;

C. 1, 2, 4;

D. 1, 3, 5;

E. 1, 2, 3, 5.

Correct answer: C

Question 45 - PES Cardiology, Spring Session 2025

To reduce the risk of hospitalization due to HF, finerenone should be used in patients:

A. with HFpEF;

B. with HFrEF;

C. with diabetes;

D. with diabetes, eGFR >60 mL/min/1.73 m² and UACR 300-500 mg/g;

E. with diabetes, eGFR >60 mL/min/1.73 m² regardless of albuminuria severity.

Correct answer: D

Question 46 - PES Cardiology, Spring Session 2025

A 60-year-old patient was admitted for elective coronary angiography. Two months earlier, he was hospitalized due to his first ever HF decompensation following an AF attack. Since then, he has been on DOAC anticoagulation. Exercise tolerance is NYHA II/III, without angina. Concomitant diseases: hypertension, hyperlipidemia. TTE: LVEDV 65 mm, LVESV 43 mm, no contractility abnormalities, LVEF 55%, LA 56 mm, dilated mitral annulus 45 mm, marked leaflet restriction, severe mitral regurgitation: PISA 9 mm, ERO 0.4 cm², RV 60 ml, moderate tricuspid regurgitation: PISA 7 mm, ERO 0.3 cm², RV 30 ml, RVSP 55 mmHg, dilated tricuspid annulus 41 mm, and right ventricular systolic function preserved. The ECG showed sinus rhythm of 65/min, QRS 110 ms, with no significant changes. Coronary angiography revealed mural changes. The patient was classified as:

A. conservative treatment only;

B. cardiac resynchronization therapy to reduce mitral regurgitation;

C. cardiac surgery with implantation of a prosthetic mitral valve;

D. percutaneous MitraClip implantation;

E. surgical mitral valve repair and tricuspid valve repair.

Correct answer: E

Question 47 - PES Cardiology, Spring Session 2025

A 75-year-old patient, post-CABG, was treated for COPD, stage G3b chronic kidney disease, type 2 diabetes with an HbA1C of 7.5%, and hypertension. He was hospitalized in the emergency room due to further HF decompensation. TTE revealed: LVEDV 70 mm, LVESV 48 mm, akinesia of the apical and apical segments, good contractility of the lateral basal segments, otherwise hypokinetic LV, LVEF 30%. Severe mitral regurgitation: PISA 9 mm, ERO 0.5 cm², RV 60 ml, systolic leaflet restriction, mean diastolic gradient across the valve 2 mmHg, planimetric MVA 5 cm², moderate tricuspid regurgitation, and preserved right ventricular systolic function. ECG revealed sinus rhythm with LBBB 140 ms. Coronary angiography reveals changes requiring conservative treatment. The following are recommended for patient management:

1. optimization of pharmacotherapy according to HFrEF guidelines;

2. implantation of a CRT-D device;

3. surgical mitral valve replacement;

4. surgical mitral valve repair;

5. if symptoms persist, MitraClip implantation after disqualification from cardiac surgery and TEE assessment of anatomical suitability.

The correct answer is:

A. 1, 2, 3;

B. 1, 2, 4;

C. 1, 2, 5;

D. only 3;

E. only 1.

Correct answer: C

Question 48 - PES Cardiology, Spring Session 2025

A 48-year-old woman underwent endoscopy three weeks ago due to abdominal pain and weight loss, which led to the diagnosis of gastric cancer. While awaiting an oncology panel opinion, she presented to the emergency department (ER) due to shortness of breath persisting for three days. Tests revealed: Hb = 12.1 g/dL, D-dimer = 5600 ng/ml, normal Tn and NT-proBNP, BP = 125/74 mmHg, and tachycardia = 100/min. The ER physician performed a CT angiography, confirming an embolism in the segmental arteries of the right lower lobe. In this situation, treatment should be considered:

1. dalteparin;

2. dabigatran;

3. rivaroxaban;

4. apixaban;

5. UFH.

The correct answer is:

A. only 1;

B. 1, 5;

C. 1, 3, 4;

D. 1, 2, 3, 4;

E. all of the above.

Correct answer: A

Question 49 - PES Cardiology, Spring Session 2025

A patient with breast cancer is receiving trastuzumab. After 3 months of therapy, LVEF decreases from 60% to 45%, but the patient has no HF symptoms. What is the recommended management?

1. continuation of trastuzumab;

2. immediate discontinuation of trastuzumab;

3. initiation of ACE-I/ARB and beta-blocker, followed by reassessment of LVEF;

4. diuretic therapy to prevent fluid retention;

5. switching trastuzumab to cisplatin.

The correct answer is:

A. only 1;

B. only 4;

C. only 5;

D. 1, 3;

E. 2, 3.

Correct answer: D

Question 50 - PES Cardiology, Spring Session 2025

Indicate the true statements regarding contraindications to antiplatelet therapy in patients with cancer and ACS:

1. ASA is not recommended if platelet count <10,000/µL;

2. ASA is not recommended if platelet count <30,000/µL;

3. clopidogrel is not recommended if platelet count <30,000/µL;

4. clopidogrel is not recommended if platelet count <10,000/µL;

5. prasugrel or ticagrelor is not recommended if platelet count <50,000/µL;

6. prasugrel or ticagrelor is not recommended if platelet count <30,000/µL.

The correct answer is:

A. 1, 3, 4, 5, 6;

B. 2, 4, 6;

C. 3, 6;

D. 2, 5;

E. 2, 3, 5.

Correct answer: A

Question 51 - PES Cardiology, Spring Session 2025

In a patient receiving immunosuppressive therapy with cyclosporine or tacrolimus, which DOAC is not recommended/contraindicated?

A. apixaban;

B. dabigatran;

C. edoxaban;

D. rivaroxaban;

E. none of the above.

Correct answer: B

Question 52 - PES Cardiology, Spring Session 2025

A 65-year-old woman diagnosed with right breast cancer eight months earlier, after breast-conserving surgery and completed chemotherapy and radiotherapy, underwent a chest CT scan for oncological evaluation, incidentally revealing an embolism in the right pulmonary artery, in the segmental arteries to the lower and middle lobes. The patient was urgently referred to the cardiology department. In the emergency department, cTnI and NTproBNP were measured - the results were normal, and TTE showed no signs of right ventricular overload. BP = 136/86 mmHg, heart rate 75/min, and saturation 96%. In this situation:

A. the CT angiography result may be considered false-positive and anticoagulation may be withheld;

B. anticoagulation should be initiated - optimally LMWH or any available DOAC;

C. despite PE diagnosis, according to sPESI the patient has low mortality risk and does not require hospitalization;

D. the patient should be hospitalized with intermediate-high-risk PE and treated with UFH;

E. none of the above statements is correct.

Correct answer: E

Question 53 - PES Cardiology, Spring Session 2025

A 34-year-old woman was diagnosed with intermediate-high-risk pulmonary embolism (PE) at the beginning of her third pregnancy and was treated with LMWH. This was the first episode in her life. After initiating treatment, she felt well and recently returned for a follow-up after three months of treatment. The TTE revealed no abnormalities. The pregnancy is due in one month. In this situation, you would suggest:

A. discontinuation of treatment after 3 months of effective anticoagulation;

B. discontinuation of treatment 1 day before planned delivery;

C. indefinite continuation of treatment;

D. continuation of treatment for at least 6 weeks postpartum;

E. continuation of LMWH with switch to reduced-dose apixaban postpartum.

Correct answer: D

Question 54 - PES Cardiology, Spring Session 2025

To assess patients eligible for early discharge and home treatment, the following scales can be used:

1. Pulmonary Embolism Severity Index (PESI);

2. simplified PESI (sPESI);

3. Hestia exclusion criteria;

4. HERDOO2 prediction model;

5. VTE-BLEED score.

The correct answer is:

A. all of the above;

B. 1, 2, 3;

C. 2, 3, 4;

D. 1, 2;

E. only 3.

Correct answer: B

Question 55 - PES Cardiology, Spring Session 2025

A 55-year-old woman was brought to the emergency department after fainting during physical exertion. According to her medical history, she had been experiencing shortness of breath for several days, had been treated for hypertension for 10 years, and for type 2 diabetes for 5 years. She smokes, is obese, and her father died suddenly at the age of 50. On physical examination after admission, the patient's BP was 145/74 mmHg. Tachycardia of 110/min was also noted, and the ECG showed a new RBBB. Arterial SpO2 was 89%. Physically, there were no signs of pulmonary congestion. In the following minutes, the patient's condition began to deteriorate rapidly, with increasing shortness of breath, altered consciousness, and BP dropping to 100/50 mmHg. In this clinical situation, to establish a diagnosis, the first steps should be:

A. perform coronary angiography;

B. urgently perform CT angiography of the chest to exclude PE and aortic dissection;

C. urgently perform TTE to assess RV overload and exclude other causes of shock such as tamponade or aortic dissection;

D. measure D-dimers, troponin, and NT-proBNP and proceed accordingly;

E. urgently perform chest X-ray to exclude pulmonary congestion.

Correct answer: C

Question 56 - PES Cardiology, Spring Session 2025

In a patient with a second intermediate-risk pulmonary embolism, you want to start a DOAC. Indicate the correct treatment options, according to EHRA 2021:

1. apixaban 2x10 mg for 7 days, then 2x5 mg for 3-6 months without dose reduction, then 2x2.5 mg for long-term secondary prevention;

2. apixaban 2x10 mg for 7 days, then 2x5 mg for 3-6 months with dose reduction as for AF, then 2x2.5 mg for long-term secondary prevention;

3. apixaban 2x5 mg for 7 days, then 2x2.5 mg for 3-6 months and for long-term secondary prevention without dose reduction;

4. rivaroxaban 2x15 mg for 3 weeks, then 1x20 mg without dose reduction for 3-6 months, then 1x10 mg for secondary prevention;

5. rivaroxaban 2x15 mg for 3 weeks, then 1x20 mg with dose reduction as in AF for 3-6 months, then 1x15 mg for secondary prevention;

6. dabigatran 2x150 mg after previously using a therapeutic dose of LMWH for 5 days, without dose reduction for 3-6 months, then 2x110 mg for long-term secondary prevention;

7. dabigatran 2x150 mg after previously using a therapeutic dose of LMWH for 5 days, with dose reduction as in AF for 3-6 months, then 2x110 mg for long-term secondary prevention;

8. dabigatran 2x150 mg after previous administration of a therapeutic dose of LMWH for 5 days, without dose reduction for 3-6 months, then in long-term secondary prevention 2x150 mg.

The correct answer is:

A. 2, 5, 7;

B. 1, 4, 8;

C. 3, 4, 6;

D. 2, 5, 8;

E. 3, 4, 8.

Correct answer: B

Question 57 - PES Cardiology, Spring Session 2025

In a 46-year-old woman diagnosed with pulmonary arterial hypertension of unknown etiology based on right heart catheterization, with severe HF symptoms … Apart from targeted pulmonary therapy, which treatment should be initiated?

A. SGLT2 inhibitor;

B. ARNI/ARB or ACE-I;

C. beta-blocker;

D. answers A, B, C are correct;

E. none of the above.

Correct answer: E

Question 58 - PES Cardiology, Spring Session 2025

Indicate the false statement regarding Eisenmenger syndrome:

A. it includes all large intracardiac and extracardiac shunts initially left-to-right that later reverse due to increased pulmonary vascular resistance;

B. if there is no significant hemoptysis, oral anticoagulation is recommended;

C. compared with idiopathic PAH, disease course is usually more stable;

D. bosentan is recommended in symptomatic patients to improve exercise capacity;

E. routine phlebotomy is not recommended.

Correct answer: B

Question 59 - PES Cardiology, Spring Session 2025

A 30-year-old woman was evaluated for dyspnea by TTE, which revealed a high probability of pulmonary hypertension. A cardiopulmonary exercise test (ERG) was performed next, revealing a ventilatory efficiency index (VE/VCO2) of 58, and a baseline PaCO_2_ in end-tidal air (PET CO2) of 32 mmHg, which decreased by 3 mmHg during exercise. The patient underwent right heart catheterization, revealing an elevated mPAP of 36 mmHg, a low PAWP of 9 mmHg, and a reduced cardiac index of 2.1 L/min/m² (PVR of 12.8 WU). The above results may suggest the diagnosis of:

A. pulmonary arterial hypertension;

B. PH related to lung disease and/or hypoxia;

C. PH related to chronic pulmonary artery obstruction;

D. may indicate both PAH and PH due to chronic pulmonary artery obstruction;

E. may suggest all of the above.

Correct answer: D

Question 60 - PES Cardiology, Spring Session 2025

Indicate true statements regarding management of PH associated with interstitial lung disease:

1. riociguat may be considered in PH due to idiopathic interstitial pneumonia;

2. inhaled treprostinil may be considered;

3. ambrisentan may be considered in PH due to idiopathic pulmonary fibrosis;

4. sildenafil may be considered in milder forms of PH due to interstitial lung disease.

The correct answer is:

A. 1, 2, 3, 4;

B. 2, 4;

C. 1, 3;

D. only 2;

E. only 4.

Correct answer: D

Question 61 - PES Cardiology, Spring Session 2025

A 54-year-old man presented to the clinic with worsening exercise tolerance. TTE revealed moderate tricuspid regurgitation with TRVmax = 3.3 m/s, normal right ventricle with normal systolic function, right atrium = 16 cm², mild pulmonary regurgitation with AcT = 125 ms. Pulmonary trunk = 27 mm, inferior vena cava = 20 mm, and normal respiratory movement.

Based on the echocardiographic findings, the probability of pulmonary hypertension is:

A. low;

B. intermediate;

C. high;

D. very high;

E. insufficient data to assess.

Correct answer: B

Question 62 - PES Cardiology, Spring Session 2025

In which of the following situations would you consider surgical treatment for coarctation of the aorta?

1) a patient with arterial hypertension (AH) and an invasive pressure gradient between the upper and lower arteries of 32 mmHg;

2) a patient with AH and a gradient across the aortic isthmus of 24 mmHg on TEE;

3) a patient without AH and a pressure difference between the upper and lower arteries of 35 mmHg on sphygmomanometer;

4) a patient without AH and an invasive pressure difference between the upper and lower arteries of 35 mmHg;

5) a patient with AH, an invasive pressure gradient between the upper and lower arteries of 15 mmHg, and aortic isthmus stenosis of 55% of its diameter at the level of the diaphragm.

The correct answer is:

A. all of the above;

B. 1, 2, 4, 5;

C. 1, 2, 5;

D. 1, 4, 5;

E. only 1.

Correct answer: D

Question 63 - PES Cardiology, Spring Session 2025

Indicate the true statements regarding the management of acute aortic syndrome (AAS):

1) the initial imaging test for suspected AAS is TTE;

2) in unstable patients with suspected AAS, a CT scan of the aorta or TEE should be performed;

3) if the probability of AAS is low, a low d-dimer level should be considered as a rule-out for AAS;

4) in patients with a high probability of aortic dissection, d-dimer testing is not recommended;

5) the treatment of choice for a Stanford type A aortic dissection is urgent surgery.

The correct answer is:

A. 1, 2;

B. 1, 2, 3;

C. 1, 3, 4;

D. 1, 2, 3, 4;

E. all of the above.

Correct answer: E

Question 64 - PES Cardiology, Spring Session 2025

Identify the false statement(s) regarding the management of aortic disease during pregnancy according to the 2018 ESC/PTK Guidelines:

1) a bicuspid aortic valve and an ascending aortic diameter of 51 mm constitute a contraindication to pregnancy;

2) in patients with an ascending aorta of 40-45 mm, cesarean delivery should be considered as the preferred option;

3) MRI (without gadolinium) is recommended for imaging in pregnant women with a dilated aortic arch or descending aorta;

4) in patients with Marfan syndrome and other hereditary thoracic aortic diseases, beta-blocker therapy should be considered throughout pregnancy;

5) in cases of uncomplicated type B aortic dissection, conservative treatment, including close BP monitoring, is recommended.

The correct answer is:

A. 1, 4, 5;

B. 2, 5;

C. 1, 3, 5;

D. only 2;

E. only 5.

Correct answer: D

Question 65 - PES Cardiology, Spring Session 2025

In which of the following patients can definite infective endocarditis be diagnosed?

A. patient with dual-chamber pacemaker and isolated E. faecalis from a single blood culture…;

B. patient with BAV, fever 38.5°C, high RF titer and Coxiella burnetii isolated from a single blood culture;

C. IV drug user, temperature 37.8°C, normal TTE, two blood cultures with S. epidermidis;

D. patient 3 weeks post-TAVI with fever and negative blood cultures, moderate paravalvular leak on TTE;

E. none of the above.

Correct answer: B

Question 66 - PES Cardiology, Spring Session 2025

Which of the following cannot be a minor Duke criterion?

A. body temperature 38.3°C;

B. hypertrophic cardiomyopathy;

C. Roth spots;

D. newly detected systolic murmur;

E. painful purplish nodules with ulceration on the palms.

Correct answer: D

Question 67 - PES Cardiology, Spring Session 2025

Annulus reversus refers to:

A. changes in atrioventricular annular motion in congenital heart disease;

B. changes in the native aortic annulus after TAVI;

C. altered tissue Doppler velocities of the mitral annulus seen in constrictive pericarditis;

D. a congenital defect involving the mitral annular saddle shape;

E. a pattern of GLS distribution seen e.g. in Takotsubo cardiomyopathy.

Correct answer: C

Question 68 - PES Cardiology, Spring Session 2025

Indicate the false statement regarding treatment of pericarditis:

A. colchicine dosing depends on body weight and it has the longest duration in relapse prevention;

B. if the patient takes ASA for comorbidities, it should be preferred without adding other NSAIDs;

C. indomethacin may be used in recurrent pericarditis and its dose should be slowly increased;

D. in a woman at 28 weeks of pregnancy with acute pericarditis, ASA is the first-line drug;

E. corticosteroids are usually second-line therapy in recurrent pericarditis.

Correct answer: D

Question 69 - PES Cardiology, Spring Session 2025

Antibiotics used in initial empirical therapy of native-valve infective endocarditis (not healthcare-associated) include:

1. cloxacillin;

2. meropenem;

3. gentamicin;

4. rifampicin;

5. daptomycin;

6. ceftriaxone.

The correct answer is:

A. 1, 2, 3;

B. 2, 3, 6;

C. 1, 3, 6;

D. 4, 5, 6;

E. 1, 4, 5.

Correct answer: C

Question 70 - PES Cardiology, Spring Session 2025

In a patient treated for native aortic valve infective endocarditis (NYHA III), control TTE shows aortic regurgitation with PHT 190 ms, holodiastolic flow reversal in the descending aorta (end-diastolic velocity 25 cm/s), and LVEF 50%. The most appropriate management is:

A. surgery within 24 hours;

B. surgery within 3-5 days;

C. surgery within 7-10 days;

D. non-urgent surgery during the same hospitalization;

E. targeted antibiotic therapy with observation.

Correct answer: B

Question 71 - PES Cardiology, Spring Session 2025

An 80-year-old man plans dental scaling. Which antibiotic should not be used for infective endocarditis prophylaxis?

A. ceftriaxone;

B. clarithromycin;

C. amoxicillin;

D. clindamycin;

E. scaling does not require antibiotic prophylaxis.

Correct answer: D

Question 72 - PES Cardiology, Spring Session 2025

Which of the following do not belong to minor diagnostic criteria of infective endocarditis?

1. presence of rheumatoid factor;

2. spondyloarthritis;

3. intracranial hemorrhagic lesions;

4. glomerulonephritis;

5. elevated ANA2 titer.

The correct answer is:

A. 1, 3;

B. only 2;

C. only 3;

D. only 4;

E. only 5.

Correct answer: E

Question 73 - PES Cardiology, Spring Session 2025

After ischemic stroke in the course of infective endocarditis, it is recommended to:

A. delay cardiac surgery for at least one month;

B. disqualify the patient from surgery;

C. always perform immediate surgery;

D. perform immediate surgery if HF, uncontrolled infection, abscess, or high embolic risk are present, provided there is no coma and intracranial hemorrhage is excluded;

E. perform immediate surgery even with intracranial hemorrhage.

Correct answer: D

Question 74 - PES Cardiology, Spring Session 2025

When suspecting infective endocarditis after TAVI, one should consider that:

1. vegetations on valve leaflets confirm diagnosis;

2. up to 60% of cases show no vegetations on TEE, so FDG-PET/CT should be used;

3. the valve frame should be assessed, as vegetations are located there in 12-19% of cases;

4. the clinical course is often atypical, including absence of fever;

5. in one-third of cases, vegetations are located outside the implanted valve, mainly on the mitral valve.

The correct answer is:

A. only 1;

B. only 2;

C. only 3;

D. only 4;

E. all of the above.

Correct answer: E

Question 75 - PES Cardiology, Spring Session 2025

Indicate true statements regarding consequences of significant chronic aortic regurgitation:

1. long-term volume and pressure overload leads to increased diastolic pressure in the LV and aorta;

2. in the compensated phase, LV volume and wall thickness increase with eccentric hypertrophy and preserved LVEF;

3. long-term pressure overload reduces compliance and increases filling pressure, leading to LV dilation and reduced EF;

4. long-term overload leads to decreased systolic pressure in the LV and aorta;

5. compensated phase involves concentric hypertrophy with reduced LV volume.

The correct answer is:

A. 1, 5;

B. 2, 3;

C. 3, 4;

D. 2, 4;

E. 4, 5.

Correct answer: B

Question 76 - PES Cardiology, Spring Session 2025

Based on the presented clinical data, the correct diagnosis is:

A. DCM with severe mitral regurgitation;

B. HCM with severe mitral regurgitation;

C. HCM with LVOTO and moderate mitral regurgitation;

D. non-dilated LV cardiomyopathy with moderate mitral regurgitation;

E. HCM with LVOTO and severe mitral regurgitation.

Correct answer: C

Question 77 - PES Cardiology, Spring Session 2025

In severe primary mitral regurgitation (PMR):

1. surgery is recommended in symptomatic patients who are eligible for surgery and are not at high perioperative risk;

2. surgery is recommended in asymptomatic patients with LV dysfunction (LVESD ≥40 mm and/or LVEF ≤60%);

3. mitral valve repair should be considered in asymptomatic low-risk patients with LVEF >60%, LVESD <40 mm, and significant LA enlargement (volume index ≥60 ml/m² or diameter ≥55 mm), provided the procedure is performed in a reference center and durable repair is likely;

4. surgery should not be considered in asymptomatic patients with preserved LV function (LVESD 60%) and AF secondary to mitral regurgitation or pulmonary hypertension (resting SPAP >50 mmHg);

5. valve replacement is the recommended surgical technique in every case.

The correct answer is:

A. 1, 3, 4;

B. 1, 2, 3;

C. 1, 2, 5;

D. 1, 2, 4;

E. 2, 3, 4.

Correct answer: B

Question 78 - PES Cardiology, Spring Session 2025

Which of the following is not an echocardiographic criterion of severe primary mitral regurgitation (PMR)?

A. flail mitral leaflet;

B. eccentric regurgitant jet of variable size impinging on the wall;

C. ERO ≥40 mm², regurgitant volume ≥60 ml, regurgitant fraction ≥50%;

D. normal LV and left atrial dimensions;

E. systolic flow reversal in the pulmonary veins.

Correct answer: D

Question 79 - PES Cardiology, Spring Session 2025

A 75-year-old patient underwent dobutamine stress echocardiography due to suspected severe low-flow, low-gradient aortic stenosis. Resting measurements showed: LVEF = 25%, LVSVi = 27 ml/m², AVmax = 3.17 m/s, peak gradient 40 mmHg, mean gradient 22 mmHg, AVA by continuity equation 0.8 cm², DVI 0.18. At peak stress the following parameters were obtained: LVEF 35%, LVSVi 49 ml/m², peak velocity 4.7 m/s, peak gradient 88 mmHg, mean gradient 57 mmHg, AVA 0.9 cm², DVI 0.22. What conclusions can be drawn from the dobutamine test?

A. the test was non-diagnostic due to lack of contractile reserve;

B. the test was unnecessary because severe aortic stenosis could already be diagnosed at rest;

C. left ventricular contractile reserve and severe aortic stenosis were confirmed;

D. left ventricular contractile reserve was confirmed and severe aortic stenosis was excluded;

E. answers A and B are correct.

Correct answer: C

Question 80 - PES Cardiology, Spring Session 2025

In patients with asymptomatic severe aortic stenosis, intervention is indicated or may be considered:

1. regardless of left ventricular ejection fraction (LVEF);

2. when LVEF <50% without another cause;

3. when LVEF <55% without another cause;

4. when LVEF >55%, procedural risk is low, and AVmax >5 m/s;

5. when LVEF >55%, procedural risk is low, and BNP level is elevated >2 times the upper limit of normal.

The correct answer is:

A. only 1;

B. 2, 3, 4;

C. 2, 3, 4, 5;

D. only 2;

E. only 4.

Correct answer: B

Question 81 - PES Cardiology, Spring Session 2025

Severe tricuspid regurgitation can be diagnosed based on echocardiographic parameters of:

1. EROA ≥40 mm²;

2. EROA ≥30 mm²;

3. regurgitant volume ≥45 mL;

4. regurgitant volume ≥60 mL;

5. dominant tricuspid inflow E-wave with velocity ≥1 m/s.

The correct answer is:

A. 1, 4;

B. 2, 4;

C. 1, 3, 5;

D. only 5;

E. 2, 4, 5.

Correct answer: C

Question 82 - PES Cardiology, Spring Session 2025

Indicate the false statement regarding management in the integrated assessment of low-gradient aortic stenosis with AVA = 0.8 cm² and mean transvalvular gradient = 35 mmHg:

A. additional parameters including stroke volume index (SVi) and LVEF should be considered to guide proper decision-making;

B. if SVi >35 ml/m² BSA, severe aortic stenosis is unlikely;

C. if SVi ≤35 ml/m² BSA and LVEF <50%, severe aortic stenosis should be diagnosed;

D. if SVi ≤35 ml/m² BSA and LVEF ≥50%, an integrated approach including CT assessment of aortic valve calcification should be considered;

E. if SVi ≤35 ml/m² BSA and LVEF <50%, flow reserve should be assessed using dobutamine stress echocardiography to differentiate severe from pseudo-severe aortic stenosis.

Correct answer: C

Question 83 - PES Cardiology, Spring Session 2025

Indicate the true statement:

A. pacemaker-mediated tachycardia (PMT) indicates pacemaker malfunction;

B. fascicular tachycardia most commonly presents with LBBB morphology and is sensitive to verapamil;

C. in typical atrial flutter, positive F-waves dominate in inferior leads and negative in V1 and aVL;

D. regular QRS complexes during atrial fibrillation with ventricular rate 200/min suggests pre-excitation;

E. the presence of abandoned leads is an absolute contraindication to MRI.

Correct answer: D

Question 84 - PES Cardiology, Spring Session 2025

Indicate the correct ECG diagnostic criteria:

1. right ventricular hypertrophy: R wave in aVR ≥1.0 mV (10 mm);

2. suspected myocardial infarction during pacing: QRS complex with QR configuration in leads I, aVL, V3-V4;

3. suspected myocardial infarction in conducted rhythms with LBBB: regression of R waves (≥3 mm) in V1-V4;

4. suspected reinfarction: reappearance of ST elevation ≥0.1 mV (1 mm) in leads with previous ST changes or new Q waves.

The correct answer is:

A. all of the above;

B. 3, 4;

C. 2, 3;

D. only 4;

E. 1, 2.

Correct answer: B

Question 85 - PES Cardiology, Spring Session 2025

Indicate the correct ECG diagnostic criteria:

1. left ventricular hypertrophy: S in V3 + R in aVL >2.8 mV (28 mm) in men;

2. LV hypertrophy with left anterior hemiblock: S in III + maximal QRS amplitude (R+S from one precordial lead) >2.8 mV (28 mm) in women;

3. LV hypertrophy with RBBB: R in lead I >1.1 mV (11 mm);

4. right ventricular hypertrophy: R in V1 + S in V5 or V6 >1.05 mV (10.5 mm);

5. right ventricular hypertrophy: R in V1 ≥0.7 mV (7 mm).

The correct answer is:

A. 1, 5;

B. 1, 3, 5;

C. 2, 3, 4, 5;

D. 1, 4, 5;

E. all of the above.

Correct answer: E

Question 86 - PES Cardiology, Spring Session 2025

Which ECG finding in an asymptomatic athlete with a negative family history requires further evaluation?

A. LBBB;

B. incomplete RBBB;

C. early repolarization;

D. ectopic atrial rhythms;

E. second-degree AV block type I (Wenckebach).

Correct answer: A

Question 87 - PES Cardiology, Spring Session 2025

Indicate the true statements:

1. pseudofusion beats have QRS morphology identical to intrinsic beats;

2. type I Brugada ECG pattern (“shark fin”) includes a positive, sharp T wave;

3. in early repolarization syndrome, typical ECG changes are located in inferior and/or lateral leads;

4. bidirectional VT occurs in catecholaminergic VT, Andersen-Tawil syndrome, and digitalis intoxication.

The correct answer is:

A. all of the above;

B. 1, 3, 4;

C. 2, 3;

D. 1, 3;

E. 1, 4.

Correct answer: B

Question 88 - PES Cardiology, Spring Session 2025

Indicate the true statement(s) regarding ECG changes:

1. ST depression in right precordial leads is typical for hypervagotonia;

2. ST elevation in inferior leads with higher elevation in lead III than II is typical for proximal RCA occlusion and right ventricular infarction;

3. ST elevation in V1 and aVR indicates circumflex artery (Cx) occlusion;

4. diffuse ST elevation in multiple leads with accompanying PR depression is typical for left main coronary artery occlusion;

5. diffuse ST elevation with negative T waves and QT prolongation is typical for Takotsubo cardiomyopathy.

The correct answer is:

A. only 2;

B. 2, 5;

C. 1, 2, 5;

D. 2, 3, 4;

E. 1, 5.

Correct answer: B

Question 89 - PES Cardiology, Spring Session 2025

In which patient would you diagnose LV hypertrophy based on ECG?

A. R in V5 = 2.0 mV (20 mm) with RBBB;

B. R in lead I = 1.0 mV (10 mm) with RBBB;

C. S in III + highest QRS amplitude in precordial leads = 2.6 mV (26 mm) with LAH;

D. S in V1 + R in V5 = 4.0 mV (40 mm) with LBBB;

E. S in V2 + S in V3 = 5.8 mV (58 mm) with LBBB.

Correct answer: A

Question 90 - PES Cardiology, Spring Session 2025

Diagnostic criteria for suspected ACS in conducted rhythms with LBBB include:

A. ST elevation ≥0.1 mV (1 mm) in leads with positive QRS;

B. ST depression ≥0.5 mV (5 mm) in V1-V3;

C. ST elevation ≥0.1 mV (1 mm) in leads with negative QRS;

D. ST depression ≥0.1 mV (1 mm) in leads with R <1.0 mV (10 mm);

E. ST elevation ≥1.0 mV (10 mm) in leads with negative QRS.

Correct answer: A

Question 91 - PES Cardiology, Spring Session 2025

A 49-year-old patient with resistant hypertension, no other risk factors, currently taking maximum doses of telmisartan and torasemide, presents for exclusion of secondary hypertension. Average home BP: 140-150/85-95 mmHg, similar values in ABPM and office measurements. Persistent hypokalemia with normal renal function suggests possible primary hyperaldosteronism. To prepare the patient for hormonal testing, indicate the combination of medications neutral for RAAS assessment that you would temporarily prescribe:

A. amlodipine + bisoprolol + doxazosin;

B. verapamil/diltiazem + doxazosin + moxonidine;

C. bisoprolol + eplerenone/spironolactone + torasemide;

D. amlodipine + verapamil/diltiazem + indapamide;

E. perindopril + indapamide + amlodipine.

Correct answer: B

Question 92 - PES Cardiology, Spring Session 2025

According to ESC/PTK 2023 diabetes guidelines, indicate the true statements regarding antiplatelet/anticoagulant therapy in patients with type 2 diabetes:

1. in diabetic patients who tolerated DAPT without bleeding complications, prolongation of DAPT beyond 12 months after ACS up to 3 years should be considered;

2. in patients with diabetes and CCS or symptomatic PAD without high bleeding risk, addition of low-dose rivaroxaban (2×2.5 mg) to ASA should be considered for long-term prevention of major vascular events;

3. in patients with ACS or CCS and diabetes undergoing PCI and requiring anticoagulation, prolongation of triple therapy with low-dose ASA, clopidogrel, and DOAC up to 1 month should be considered if thrombotic risk outweighs bleeding risk;

4. in patients with ACS or CCS and diabetes undergoing PCI and requiring anticoagulation, prolongation of triple therapy with ASA, clopidogrel, and DOAC for 3 months is recommended.

The correct answer is:

A. 1, 2, 3;

B. 2, 3;

C. 1, 4;

D. only 4;

E. only 1.

Correct answer: A

Question 93 - PES Cardiology, Spring Session 2025

Which of the following data are not included in the diagnostic criteria for familial hypercholesterolemia according to the Dutch Lipid Clinic Network (DLN)?

1. untreated LDL cholesterol levels;

2. presence of tendon xanthomas or corneal arcus before age 45;

3. presence of mutations in LDL receptor or apolipoprotein B genes;

4. presence of mutations in the lipoprotein lipase gene;

5. presence of mutations in the PCSK9 gene;

6. family history of premature vascular disease (men <55 years; women <60 years) in a first-degree relative.

The correct answer is:

A. 1, 5;

B. 2, 5;

C. 3, 5;

D. only 4;

E. all of the above are DLN diagnostic criteria.

Correct answer: D

Question 94 - PES Cardiology, Spring Session 2025

In which case does ESC 2021 recommend the use of ASA for primary prevention?

A. in all patients over 50 years of age;

B. in patients with type 2 diabetes without additional risk factors;

C. in patients with high cardiovascular risk and low bleeding risk;

D. in patients with arterial hypertension to reduce stroke risk;

E. ASA is not recommended for primary prevention.

Correct answer: C

Question 95 - PES Cardiology, Spring Session 2025

Indicate the incorrect recommendation regarding dyslipidemia treatment in patients with moderate to severe chronic kidney disease (CKD):

A. statins or statin-ezetimibe combination are recommended in non-dialysis CKD stage 3-5;

B. in patients with ASCVD already treated with statins, ezetimibe, or their combination at dialysis initiation, continuation should be considered;

C. in CKD patients without ASCVD requiring dialysis, initiation of statin therapy is not recommended;

D. in CKD patients with ASCVD, fibrates are preferred due to hepatic elimination;

E. benefits of PCSK9 inhibitors may extend to patients with earlier CKD stages (eGFR 60-90 and 30-60 ml/min/1.73 m²).

Correct answer: D

Question 96 - PES Cardiology, Spring Session 2025

Indicate the false statement(s) regarding treatment of type 2 diabetes in patients with heart failure:

1. DPP-4 inhibitors (sitagliptin and linagliptin) have a neutral effect on hospitalization risk and should be considered in diabetic patients at risk of or with HF;

2. pioglitazone is associated with increased HF risk and is not recommended in patients at risk of HF;

3. the DPP-4 inhibitor saxagliptin is associated with reduced HF hospitalization risk and should be preferred in patients at risk of HF;

4. switching glucose-lowering therapy from agents without proven CV benefit or safety to agents with proven benefit is recommended;

5. basal insulins (glargine and degludec) have neutral effects on HF hospitalization risk and should be considered in diabetic patients at risk of or with HF.

The correct answer is:

A. 2, 4;

B. 2, 3;

C. only 3;

D. 3, 5;

E. 1, 5.

Correct answer: C

Question 97 - PES Cardiology, Spring Session 2025

Indicate correct recommendations for patients with diabetes:

1. finerenone is recommended as add-on therapy to ACEI or ARB in patients with eGFR >60 mL/min/1.73 m² and UACR ≥30 mg/mmol (≥300 mg/g) or eGFR 25-60 mL/min/1.73 m² and UACR ≥3 mg/mmol (≥30 mg/g) to reduce cardiovascular and renal risk;

2. GLP-1 receptor agonists are recommended for glycemic control at eGFR >15 mL/min/1.73 m² due to low hypoglycemia risk and beneficial effects on body weight and albuminuria;

3. SGLT2 inhibitors (canagliflozin, empagliflozin, or dapagliflozin) are recommended in CKD patients with eGFR ≥20 mL/min/1.73 m² to reduce cardiovascular and renal risk;

4. in patients with CKD and ASCVD, routine high-dose proton pump inhibitor therapy is recommended due to high GI bleeding risk;

5. in patients with CKD and ASCVD, low-dose ASA 75-100 mg once daily is recommended;

6. in patients with chronic symptomatic peripheral artery disease without high bleeding risk, rivaroxaban 2×2.5 mg should be used as an alternative to ASA 75-100 mg.

The correct answer is:

A. 1, 2, 3, 4;

B. 1, 2, 3, 5;

C. 1, 2, 3, 6;

D. 2, 3, 4, 5;

E. 3, 4, 5, 6.

Correct answer: B

Question 98 - PES Cardiology, Spring Session 2025

Indicate the true statements regarding bempedoic acid:

1. it reduces cholesterol synthesis with very limited musculoskeletal adverse effects;

2. it lowers LDL cholesterol as monotherapy and shows synergy with ezetimibe;

3. it may be used in statin-intolerant patients as monotherapy or combined with ezetimibe;

4. it can be combined with statins;

5. in statin-intolerant patients at high CV risk, it resulted in a significant reduction in the composite of death, non-fatal MI, non-fatal stroke, and coronary revascularization.

The correct answer is:

A. 1, 2, 3, 5;

B. 1, 2, 4, 5;

C. 1, 2, 4;

D. 1, 2, 3;

E. all of the above.

Correct answer: E

Question 99 - PES Cardiology, Spring Session 2025

Which of the following are not internal causes of bradycardia?

A. myocardial infarction, ischemia;

B. cardiomyopathy;

C. degenerative diseases of the impulse-forming/conduction system;

D. genetic disorders;

E. vagal reflex.

Correct answer: E

Question 100 - PES Cardiology, Spring Session 2025

An 80-year-old man underwent TAVI. Pre-procedural ECG: sinus rhythm 75 bpm, PR 180 ms, LV hypertrophy, no intraventricular conduction disturbances, QRS 100 ms. Post-procedural ECG: sinus rhythm 70 bpm, PR 180 ms, LBBB with QRS 160 ms. TTE shows no pericardial effusion, normal prosthetic valve function, LVEF 50%. What should be the next management?

A. follow-up ECG in a few days;

B. immediate EPS;

C. observation with continuous ECG monitoring or EPS no earlier than 3 days post-procedure;

D. implantation of a dual-chamber pacemaker, preferably with His bundle or left bundle branch pacing;

E. implantation of CRT-D if LBBB persists in the following days.

Correct answer: C

Question 101 - PES Cardiology, Spring Session 2025

In patients with bundle branch block (BBB), which is not an indication for pacemaker implantation?

A. unexplained syncope and bifascicular block with: HV ≥70 ms, second- or third-degree AV block, or block located at or below the His bundle during incremental atrial pacing or abnormal pharmacological stress response;

B. unexplained syncope and bifascicular block without EPS in elderly, frail patients with high risk and recurrent syncope;

C. alternating BBB regardless of symptoms;

D. bifascicular block in asymptomatic patients;

E. new-onset LBBB and HV interval 80 ms in post-TAVI patients.

Correct answer: D

Question 102 - PES Cardiology, Spring Session 2025

His bundle pacing (HBP) should be used or considered in:

A. patients with sinus node disease without AV conduction disturbances and expected low ventricular pacing burden;

B. patients with distal AV conduction disturbances and prolonged HV interval, without backup RV lead;

C. patients with AV block and LVEF >40% with expected ventricular pacing >20%, HBP may be considered;

D. asymptomatic bifascicular block;

E. cardioinhibitory vasovagal syncope.

Correct answer: C

Question 103 - PES Cardiology, Spring Session 2025

Permanent pacing should be considered in patients after acute myocardial infarction:

A. if complete AV block does not resolve within at least 5 days after MI;

B. if new-onset LBBB occurs regardless of bradycardia symptoms;

C. if AV block resolves spontaneously or after revascularization;

D. in bifascicular block regardless of bradycardia symptoms;

E. in asymptomatic sinus bradycardia.

Correct answer: A

Question 104 - PES Cardiology, Spring Session 2025

Pacemaker implantation after TAVI is indicated in the case of:

A. prophylactic permanent pacing before TAVI in patients with RBBB and no pacing indication;

B. new-onset, alternating bundle branch block after TAVI;

C. new-onset LBBB with QRS >150 ms without further progression of conduction disturbances >48 h after TAVI if EPS shows HV <70 ms;

D. new-onset LBBB with QRS >150 ms without further progression of conduction disturbances >48 h after TAVI even if ambulatory ECG monitoring shows no AV conduction abnormalities;

E. pacemaker implantation before TAVI in all patients due to high incidence of AV conduction disturbances.

Correct answer: B

Question No. 105 - PES Cardiology, Spring Session 2025

According to the ESC guidelines, which of the following tests is most helpful in the diagnosis of arrhythmogenic cardiomyopathy (ACM)?

A. resting ECG;

B. exercise stress test;

C. cardiac magnetic resonance (CMR);

D. 24-hour Holter ECG;

E. echocardiography.

Correct answer: C

Question No. 106 - PES Cardiology, Spring Session 2025

Which management would be considered incorrect in a 52-year-old woman with exertional dyspnea NYHA class III, in whom TTE showed interventricular septal thickness of 21 mm, an LVOT gradient of 60 mmHg, and no other causes of symptoms?

A. alcohol septal ablation to reduce septal thickness;

B. surgical septal myectomy;

C. use of digoxin;

D. use of a β-blocker or verapamil;

E. electrical cardioversion and ablation if atrial fibrillation occurs.

Correct answer: C

Question No. 107 - PES Cardiology, Spring Session 2025

In hypertrophic cardiomyopathy with LVOT obstruction (HCM with LVOTO), it is recommended to:

1. treat with a non-vasodilating beta-blocker, titrated gradually to the maximum tolerated dose;

2. treat with digoxin in case of intolerance or contraindications to beta-blockers;

3. consider ICD implantation for primary prevention when the 5-year SCD risk is >6% based on the HCM Risk-SCD calculator after detailed clinical assessment;

4. perform surgical myectomy or alcohol septal ablation to reduce symptoms in patients in NYHA class III-IV despite maximally tolerated conservative therapy;

5. when ICD implantation is indicated, consider a dual-chamber device for AV sequential pacing with AV optimization to reduce the LVOT gradient and facilitate beta-blocker or verapamil therapy.

The correct answer is:

A. only 1;

B. 1, 2, 4;

C. 1, 2;

D. 1, 3, 4, 5;

E. 2, 3.

Correct answer: D

Question No. 108 - PES Cardiology, Spring Session 2025

When cardiac amyloidosis is suspected based on TTE or CMR, the following subsequent steps are recommended:

1. scintigraphy with 99mTc-PYP/DPD/HMDP;

2. 18F-FDG PET;

3. assessment of serum free light chains and immunofixation of serum and urine proteins;

4. genetic testing if ATTR amyloidosis is suspected;

5. cardiac/extracardiac biopsy in all patients.

The correct answer is:

A. 1, 3, 4;

B. 2, 3, 4;

C. 2, 3, 5;

D. 1, 5;

E. 2, 5.

Correct answer: A

Question No. 109 - PES Cardiology, Spring Session 2025

Potential acquired causes of dilated cardiomyopathy include:

1. Trypanosoma cruzi infection;

2. use of methylphenidate in ADHD therapy;

3. use of anabolic steroids;

4. hyperthyroidism;

5. hypothyroidism;

6. primary biliary cirrhosis;

7. peripartum period;

8. ulcerative colitis.

The correct answer is:

A. 1, 3, 4, 7;

B. 1, 3, 5, 7;

C. 2, 3, 4, 6;

D. 5, 6, 7, 8;

E. All of the above.

Correct answer: E

Question No. 110 - PES Cardiology, Spring Session 2025

Warning signs (“red flags”) suggesting Anderson-Fabry disease in patients with LV hypertrophy include:

1. angiokeratomas;

2. carpal tunnel syndrome;

3. neuropathic pain;

4. proteinuria;

5. bilateral hilar lymphadenopathy.

The correct answer is:

A. 1, 2, 3;

B. 1, 2, 4;

C. 2, 3, 5;

D. 1, 3, 4;

E. Only 1.

Correct answer: D

Question No. 111 - PES Cardiology, Spring Session 2025

In the differential diagnosis of patients with HCM, specific diseases to consider include:

1. amyloidosis;

2. Chagas disease;

3. sarcoidosis;

4. hemochromatosis;

5. Anderson-Fabry disease.

The correct answer is:

A. 1, 3;

B. 2, 4;

C. 1, 5;

D. 1, 4;

E. 1, 2.

Correct answer: C

Question No. 112 - PES Cardiology, Spring Session 2025

A 40-year-old woman with HCM complains of exertional dyspnea (NYHA II/III) and palpitations. TTE shows maximal interventricular septal thickness of 20 mm and LVOT with a resting systolic gradient of 25 mmHg. Cardiac MRI shows areas of LGE >15% and LVEF 55%. The patient’s SCD risk (Risk-SCD calculator) is <4%. Propose the appropriate management:

1. optimize pharmacological therapy: beta-blocker, and if intolerance or contraindications exist, verapamil or diltiazem; if symptoms persist, consider adding mavacamten;

2. assess the degree of LVOT obstruction during the Valsalva maneuver - if the maximal gradient is 40 mmHg, consider septal reduction therapy;

3. ICD implantation may be considered in this patient, taking into account extensive LGE >15% on cardiac MRI;

4. there are no indications for ICD implantation due to low SCD risk, and LGE assessment on MRI is irrelevant for ICD decision-making;

5. given the low resting LVOT gradient and LVEF ≥50%, ACE inhibitors should be included in therapy.

The correct answer is:

A. 1, 4;

B. 2, 3;

C. 1, 3;

D. 2, 4;

E. 4, 5.

Correct answer: C

Question No. 113 - PES Cardiology, Spring Session 2025

In a 52-year-old asymptomatic patient with bicuspid aortic valve (BAV), follow-up TTE revealed aortic regurgitation with the following parameters: PISA 8 mm, ERO 0.4 cm², RV 70 ml, ascending aorta dilation 50 mm, LVESD 53 mm with LVEF 55%. Indicate the correct management:

A. continued observation - follow-up every 6-12 months;

B. aortic regurgitation parameters and aortic diameter indicate urgent surgery;

C. verification of the degree of LV dysfunction by TEE is required;

D. verification of regurgitant volume by CT before treatment decision is required;

E. elective surgical treatment.

Correct answer: E

Question No. 114 - PES Cardiology, Spring Session 2025

A pregnant woman is not at extremely high risk of death in the case of:

A. pulmonary arterial hypertension confirmed by right heart catheterization;

B. Fontan circulation with late complications;

C. severe, symptomatic mitral and aortic stenosis;

D. corrected transposition of the great arteries with preserved systolic function of the systemic ventricle;

E. aortic dilation in Turner syndrome >25 mm/m² BSA.

Correct answer: D

Question No. 115 - PES Cardiology, Spring Session 2025

Indicate the false statements regarding management of patients with secundum atrial septal defect (ASD II):

1. detection of ASD II in young patients is always an indication for percutaneous occluder implantation regardless of RV overload or pulmonary hypertension values;

2. once indications are established, the method of choice is percutaneous ASD II closure if technically feasible;

3. in patients with ASD II and LV dysfunction, balloon occlusion testing is indicated to confirm high LV filling pressures before closure;

4. in patients with Eisenmenger syndrome and severe pulmonary hypertension, defect closure is contraindicated;

5. in patients after ischemic stroke without other cardiovascular risk factors, closure of ASD II should be considered.

The correct answer is:

A. 1, 3;

B. 2, 5;

C. 3, 4;

D. 4, 5;

E. 1, 2;

Correct answer: A

Question No. 116 - PES Cardiology, Spring Session 2025

Indicate the false statement regarding patients after complete repair of tetralogy of Fallot:

A. the most common indications for reoperation are pulmonary valve regurgitation, RVOT stenosis, and reopening of a ventricular septal defect;

B. more than half of patients require reoperation after 15 years, most commonly due to pulmonary regurgitation after RVOT obstruction repair;

C. pulmonary valve replacement is recommended in all asymptomatic patients with severe pulmonary regurgitation regardless of RV volume, systolic function, or degree of tricuspid regurgitation;

D. transcatheter pulmonary valve implantation is currently preferred in patients with a non-native RV outflow tract (RVOT);

E. pulmonary valve replacement is recommended in symptomatic patients with severe pulmonary regurgitation and/or at least moderate RVOT stenosis.

Correct answer: C

Question No. 117 - PES Cardiology, Spring Session 2025

A 60-year-old patient with a childhood diagnosis of insignificant ASD II presents with several months of worsening dyspnea and lower-limb edema. TTE shows: normal LV EF and dimensions, mild mitral regurgitation, moderate tricuspid regurgitation, basal RV diameter in apical 4-chamber view 43 mm, TAPSE 14 mm, signs of RV overload, and Qp/Qs 1.6. What is the correct next step?

A. search for causes of RV damage with pulmonary CT angiography, as the septal defect was previously considered insignificant;

B. pulmonology work-up;

C. TEE with a plan for qualification for percutaneous ASD closure;

D. surgical treatment of the defect and tricuspid regurgitation;

E. defect correction is contraindicated due to its long-standing nature.

Correct answer: C

Question No. 118 - PES Cardiology, Spring Session 2025

Indicate true statements regarding Ebstein anomaly:

1. the most common associated arrhythmia is atrial flutter;

2. the most common associated arrhythmia is AVRT;

3. it often coexists with ASD;

4. it often coexists with membranous VSD;

5. ECG frequently shows pre-excitation;

6. ECG frequently shows RBBB.

The correct answer is:

A. 1, 3, 6;

B. 2, 3, 5, 6;

C. 2, 4, 5, 6;

D. 1, 4, 5, 6;

E. 1, 6.

Correct answer: B

Question No. 119 - PES Cardiology, Spring Session 2025

In a 34-year-old asymptomatic patient, TTE performed for sports medicine purposes suggested secundum ASD with enlargement of right-sided chambers, increased pulmonary flow, and mildly reduced RV systolic function. TEE confirmed ASD II with a diameter of 21 mm, tissue rims of 7 mm, an aortic rim of 3 mm, and no other shunts. What is the correct management?

A. continued observation;

B. surgical treatment without additional testing;

C. percutaneous defect closure;

D. perform CMR;

E. coronary angiography and qualification for interventional treatment.

Correct answer: C

Question No. 120 - PES Cardiology, Spring Session 2025

Indicate the false statement regarding patent ductus arteriosus (PDA):

A. it represents a persistent fetal connection between the left pulmonary artery and the descending aorta;

B. closure is not recommended in established Eisenmenger syndrome;

C. in the presence of LV volume overload without pulmonary hypertension, it is an indication for closure regardless of symptoms;

D. a large PDA presents with differential cyanosis;

E. surgical treatment is the method of choice for PDA closure.

Correct answer: E

## References

[1] Bommasani R, Hudson DA, Adeli E (2026). On the opportunities and risks of foundation models. arXiv.

[2] Thirunavukarasu AJ, Ting DS, Elangovan K, Gutierrez L, Tan TF, Ting DS (2023). Large language models in medicine. Nat Med.

[3] Kung TH, Cheatham M, Medenilla A (2023). Performance of ChatGPT on USMLE: potential for AI-assisted medical education using large language models. PLOS Digit Health.

[4] Gilson A, Safranek CW, Huang T (2022). How well does ChatGPT do when taking the medical licensing exams? The implications of large language models for medical education and knowledge assessment. medRxiv.

[5] Jaworski A, Jasiński D, Jaworski W (2024). Comparison of the performance of artificial intelligence versus medical professionals in the Polish Final Medical Examination. Cureus.

[6] Suwała S, Szulc P, Guzowski C (2024). ChatGPT-3.5 passes Poland's medical final examination-Is it possible for ChatGPT to become a doctor in Poland?. SAGE Open Med.

[7] Fuentes-Martín Á, Cilleruelo-Ramos Á, Segura-Méndez B, Mayol J (2023). Can an artificial intelligence model pass an examination for medical specialists?. Arch Bronconeumol.

[8] Demir S (2024). A comparative analysis of GPT-3.5 and GPT-4.0 on a multiple-choice ophthalmology question bank: a study on artificial intelligence developments. Rom J Ophthalmol.

[9] (2025). Medical Examination Center (CEM). National Specialization Examination. Łódź. https://www.cem.edu.pl.

[10] Latkowska A, Sawina P, Dolata T (2025). Assessment of the ability of the ChatGPT-5 model to pass the Endocrinology Specialization Exam. Cureus.

[11] Loson-Kawalec M, Kowalczyk A, Tabor A (2025). Comparison of GPT-4o with human performance in the Polish Vascular Surgery Specialty Examination. Cureus.

[12] Wielochowska A, Stachowicz A, Olender M (2025). The effectiveness of the multimodal language model, Google Gemini 2.5 Pro, in solving the Specialization Exam in Gynecology and Obstetrics. Cureus.

[13] Boczkowski D, Dolata T, Radej D (2025). Assessment of the efficacy of the Google Gemini 2.5 Pro model in solving the Polish State Specialization Exam in Pediatric Surgery. Cureus.

[14] Ros-Arlanzón P, Perez-Sempere A (2024). Evaluating AI competence in specialized medicine: comparative analysis of ChatGPT and neurologists in a neurology specialist examination in Spain. JMIR Med Educ.

[15] Sarangi PK, Narayan RK, Mohakud S, Vats A, Sahani D, Mondal H (2024). Assessing the capability of ChatGPT, Google Bard, and Microsoft Bing in solving radiology case vignettes. Indian J Radiol Imaging.

[16] Kowalczyk A, Loson-Kawalec M, Tabor A (2025). Comparison of GPT-5 responses with the official results of the Polish Specialized Psychiatric Examination in Child and Adolescent Psychiatry. Cureus.

